# Textile‐based Low‐frequency RC Filter for Noise Reduction in ECG signals

**DOI:** 10.1002/gch2.202400237

**Published:** 2025-02-11

**Authors:** Nada Al‐azzawi, Irem Yunculer, Kadir Ozlem, Munire Sibel Cetin, Asli Tuncay Atalay, Ozgur Atalay, Gökhan Ince

**Affiliations:** ^1^ Faculty of Computer and Informatics Engineering, Computer Engineering Department Istanbul Technical University Istanbul 34469 Turkey; ^2^ Faculty of Textile Technologies and Design, Textile Engineering Department Istanbul Technical University Istanbul 34437 Turkey

**Keywords:** ECG filtering, e‐textile filters, low frequency filtering, RC filters, smart shirt, textile‐based electrodes, wearable RC filter

## Abstract

Advancements in electronic textiles over the past decade have significantly transformed the field of wearable technology, with recent developments leading to the production of a wide array of textile‐based sensing and actuation systems. Beyond sensors and actuators, textile‐based technologies can benefit from the integration of additional electronic solutions within the framework of textilization. One such solution is filtering, which has primarily been explored in the context of high‐frequency applications in e‐textiles. In contrast, low‐frequency filtering has received limited attention in the literature. This study investigates the design and fabrication of low‐frequency textile‐based Resistor–Capacitor (RC) filters, emphasizing their potential for wearability. Various materials and geometric configurations are explored for the resistive and capacitive components of the filter, evaluating their performance in terms of frequency response. Additionally, these filters are integrated with textile‐based electrodes and assess their filtering efficacy at a cutoff frequency of approximately 100 Hz within the context of an electrocardiogram (ECG) application during both static and dynamic activities. The results demonstrate that textile‐based filters can serve as viable alternatives to conventional electronic filters, exhibiting comparable performance in noise suppression, as evidenced by signal‐to‐noise ratio (SNR) improvements of 25 dB during static activities and 11 dB during dynamic activities.

## Graphical abstract

Textile‐based RC filtering components are developed. The design of these components is subjected to constraints of frequency response quality and the size and tolerance potential for wearability. These filtering components are integrated into textile‐based electrodes embedded into a t‐shirt to monitor ECG. The performance of the filter is highlighted by comparing it to the performance of standard electronics in terms of SNR of the signal.

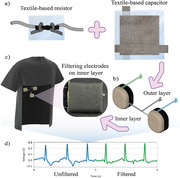

## 1 Introduction

In recent years, significant advancements in the field of wearable technology have been driven by the integration of electronic functionalities into textiles, resulting in the emergence of electronic textiles (e‐textiles). These smart fabrics are capable of sensing, reacting to, and adapting to external stimuli, laying the foundation for the development of intelligent textile systems. The parallel progress of wireless microcontrollers and lightweight batteries has facilitated the creation of wearable systems, where textiles play a central role as sensors.^[^ ^1^, ^2^, ^3^, ^4^, ^5^, ^6^, ^7^ ^]^ Over the years, a wide range of textile‐based sensors have been developed, including inductive,^[^ ^8^, ^9^ ^]^ piezoresistive,^[^ ^10^, ^11^ ^]^ resistive,^[^ ^12^, ^13^ ^]^ capacitive,^[^ ^14^, ^15^ ^]^ and piezoelectric sensors,^[^ ^16^, ^17^ ^]^ with resistive and capacitive sensors being particularly relevant to this study.

Beyond the development of sensors, e‐textile research has also increasingly focused on “textilizing” various electronic solutions, exemplified by the creation of textile‐based resonators and antennas.^[^ ^18^, ^19^ ^]^ These advancements introduce the potential for improving signal quality within wearable systems, shifting the focus from signal generation to signal transmission. Filtering, a fundamental electronic solution found in nearly all signal‐processing circuits, is critical in this context. However, filter design requires that components such as capacitors and resistors maintain stable values to ensure consistent cutoff frequencies, a challenge given the nature of sensors, which are designed to detect variations in target quantities. Unlike filters, which necessitate stable resistance and capacitance, the design of textile‐based resistive and capacitive sensors often prioritizes variability in these parameters for sensing applications such as pressure,^[^ ^20^, ^21^, ^22^ ^]^ humidity,^[^ ^23^ ^]^ temperature,^[^ ^24^ ^]^ and strain.^[^ ^25^ ^]^ Consequently, the design of a textile‐based RC filter requires a departure from these sensor‐centric approaches, demanding that the resistive and capacitive components remain constant and that techniques promoting variation, such as stretchability, be avoided.

While there is a growing body of work addressing the design of high‐frequency filters and antennas, the design of low‐frequency textile‐based filters remains underexplored. This gap arises from two key factors. First, wearable technology has largely been built upon the foundation of wireless communication, where high‐frequency antennas and filters are essential. As such, the focus has primarily been on high‐frequency solutions to enable integration and replace traditional rigid electronic components. Second, the challenge of creating textile‐based components suitable for low‐frequency filtering stems from the need for relatively large resistor and capacitor values compared to high‐frequency designs. Most textile‐based sensors, by contrast, typically operate with small values of resistance (R) and capacitance (C), often in the tens of Ωs and pF ranges, which are sufficient for their intended applications.^[^ ^21^, ^25^, ^26^, ^27^ ^]^ However, for RC filters targeting low‐frequency applications, resistance and capacitance values can range from a few Ωs and pF to several kΩ–MΩs and nF–µF, necessitating larger surface areas for the components, which complicates their integration into wearable systems.

Nevertheless, low‐frequency signals, such as those from physiological measurements like ECG^[^ ^28^ ^]^ or mechanical skeletal joints movement,^[^ ^29^ ^]^ are central to wearable applications and could benefit significantly from low‐frequency filtering. The development of textile‐based filters capable of handling such signals could enhance wearable systems by improving signal integrity and enabling higher levels of integration. Given the essential role of filtering in electronic circuits and the predominance of low‐frequency signals in wearable applications, it is crucial to explore wearable solutions for low‐frequency filtering.

To the best of our knowledge, no previous work has specifically addressed the design of low‐frequency filters for e‐textile wearable applications. Therefore, this study aims to fill that gap by proposing a novel textile‐based RC filter design, with the following contributions:

- Design of textile‐based resistive and capacitive components with relatively high values to meet the requirements of low‐frequency filtering.
- Comparison of textile materials and design strategies for resistive and capacitive components, evaluating their impact on the filter's frequency response.
- Evaluation of the wearability of textile‐based filter components, considering their integration into garments that are worn close to the skin and subjected to movement.

**Figure 1:**
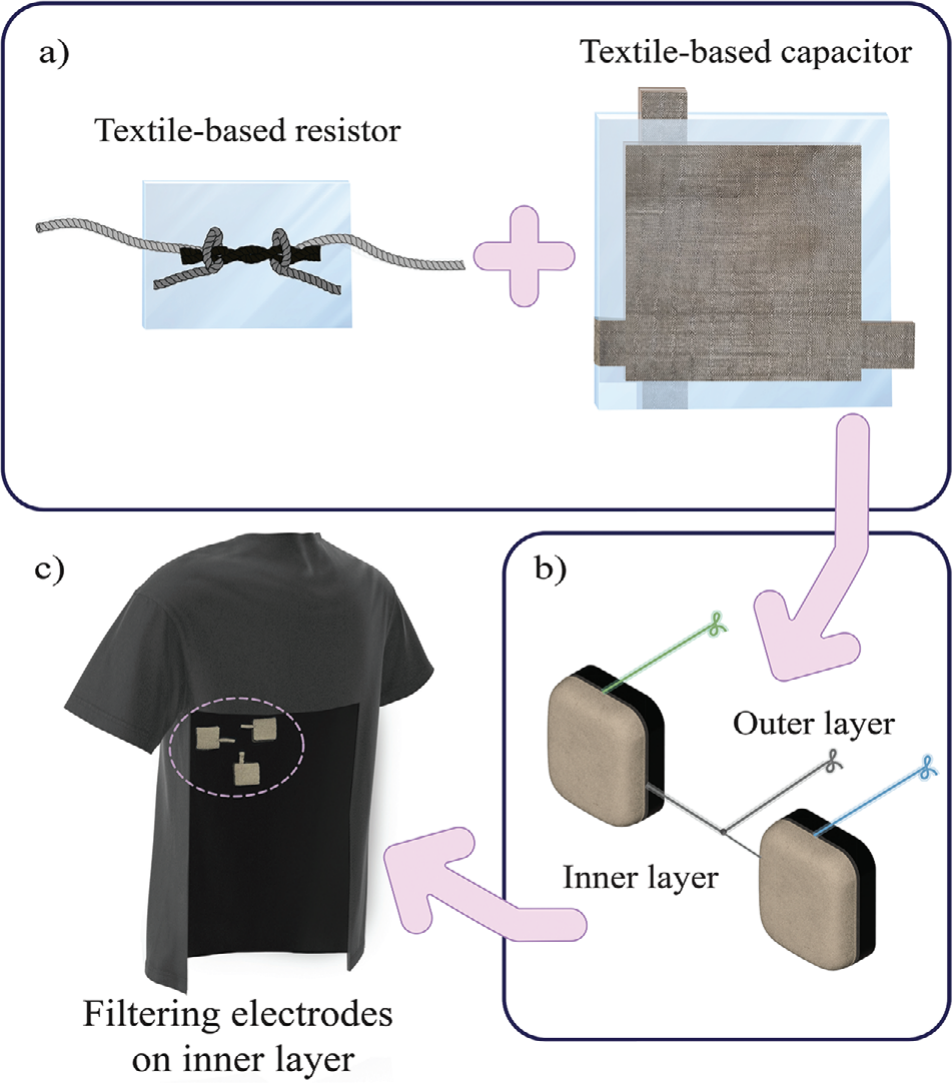
Design of the ECG measuring filtering t‐shirt system. a) The textile‐based R and C samples. b) The electrodes inside which the filtering samples will be embedded. c) The t‐shirt in which the filtering electrodes will be embedded (the t‐shirt illustration features a cut into the material for visual comprehension of the inside layer).

To assess the effectiveness of these filters, we selected ECG as the test application, enabling us to build on and compare our work with previous studies in the field. The design incorporates the textile filters into electrodes embedded in a t‐shirt, as illustrated in **Figure** **1**. The t‐shirt is composed of two distinct fabric layers: an inner layer that directly contacts the skin, and an outer layer that forms the external surface of the garment. The inner layer incorporates the electrode face through the integration of silver‐coated yarn via knitting. Positioned on the reverse side of the electrode and situated between the two fabric layers, the textile‐based filtering components are embedded, ensuring their isolation from direct skin contact.

## 2 Related Works

Filters in electronic circuits can be broadly categorized based on their intended spectral applications. These include filters designed for low‐frequency applications, as well as those intended for higher frequency ranges. Filters for higher‐frequency applications typically address the medium (300 kHz–30 MHz) and high‐frequency spectrum, extending into the ultra‐high and super‐high frequency ranges (3000 MHz–30 GHz) and beyond. A number of studies in the literature focus on the development of textile‐based high‐frequency filters. For example, Moradi et al. created a wearable meander‐line microwave band‐pass filter by embroidering conductive yarn onto a textile substrate. This filter, centered at 7.58 GHz, demonstrated high performance, with experimental results closely matching the simulated data.^[^ ^30^ ^]^ In a subsequent study, the authors introduced a textile‐based Split Ring Resonator (SRR)‐based band‐stop filter. The SRRs were embroidered onto a cotton substrate to load the transmission line, achieving effective band‐stop filtering in the 2.7–4.7 GHz range.^[^ ^31^ ^]^ Similarly, Kao et al. developed a filtering antenna by inkjet printing silver onto a textile substrate, achieving a pass‐band in the 2.5–4.6 GHz frequency range.^[^ ^32^ ^]^ Another approach by Ahmad et al. involved creating a flexible microstrip patch antenna operating at a center frequency of 5.8 GHz. The antenna patch was fabricated from conductive textile material, woven for the purpose, while the non‐conductive denim fabric served as the substrate.^[^ ^33^ ^]^

In contrast, very low‐frequency (VLF) filtering targets signals in the sub‐3 kHz range. Within the domain of e‐textile development, few studies have focused on the design of textile‐based RC filters for VLF applications. Research in this area has largely concentrated on the use of capacitive coupling in electrodes, particularly for wearable ECG applications. In this design, the electrodes are created by using conductive yarns to form a capacitive plate surface. These electrodes are mounted on a non‐conductive fabric, such as cotton, which serves as a substrate when worn. The capacitive connection is formed with the subject's skin acting as one plate of the capacitance and the conductive yarn acting as the other plate. The non‐conductive fabric in between thus acts as the dielectric layer between them. Li et al. demonstrated that such a capacitive setup could be used in conjunction with a resistor to create a high‐pass filter; however, their study did not explore the frequency response of this configuration, limiting its investigation to monitoring the quality of the output ECG signal.^[^ ^34^ ^]^

Subsequent work by Babusiak et al. explored the frequency response of a similar RC circuit by feeding the excitation signal to a copper‐plated electrode. The electrode, when put in contact with the skin, formed a full capacitance as the subject skin acted as the second capacitance plate. The capacitance value was offset by an appropriate ridged resistor to achieve a desired cutoff frequency for ECG applications. Their study compared the frequency response of the filter based on different dielectric materials, assessing how these materials affected the gain levels of the circuit.^[^ ^35^ ^]^ More recently, Terada et al.^[^ ^36^ ^]^ proposed a design that eliminated the need for a traditional rigid resistor by relying on the impedance of a capacitive electrode pressed against the skin to achieve a low‐pass filtering effect. This approach, which builds on previously explored impedance measurement techniques,^[^ ^37^, ^38^ ^]^ adjusts the capacitive component of the impedance by designing the electrode as a large, folded capacitor. Manipulating the impedance value by the increase of the capacitive component served to bring the impedance within the desired frequency range, and they were able to achieve the necessary attenuation in the reject band above 100 Hz. While their results demonstrated improved signal quality, the authors noted that designing a full textile RC filter would offer the advantage of multistage filtering, a capability that cannot be achieved by relying solely on skin contact impedance. Section S4 (Supporting Information) contains a summary table of comparison between the works found in the literature and the work proposed in this study.

## 3 Results and Discussion

### 3.1 Production of Textile‐based Resistors and Capacitors

The cutoff frequency of an RC filter is defined by the following equation:

(1) $$f_{c}=\frac{1}{2\pi RC},$$

where *f*_*c*_ is the cutoff frequency in Hz, *R* is the resistance and *C* is the capacitance. To create an RC filter with a cutoff frequency suitable for low‐frequency applications such as ECG signal noise suppression, the values of *R* and *C* must be relatively large compared to high‐frequency applications. The challenge in achieving these high values of *R* and *C* lies in the selection of textile materials and their geometrical configuration. Both factors must be optimized to achieve the desired cutoff frequency while maintaining the wearability of the components. In this study, we experimented with various materials, sizes, and fabrication techniques to produce the necessary components, targeting a cutoff frequency around 100 Hz. This frequency is particularly relevant for suppressing muscle‐originated noise in ECG signals, while the frequency components of the ECG signal itself reside below this threshold.^[^ ^36^ ^]^

**Figure 2:**
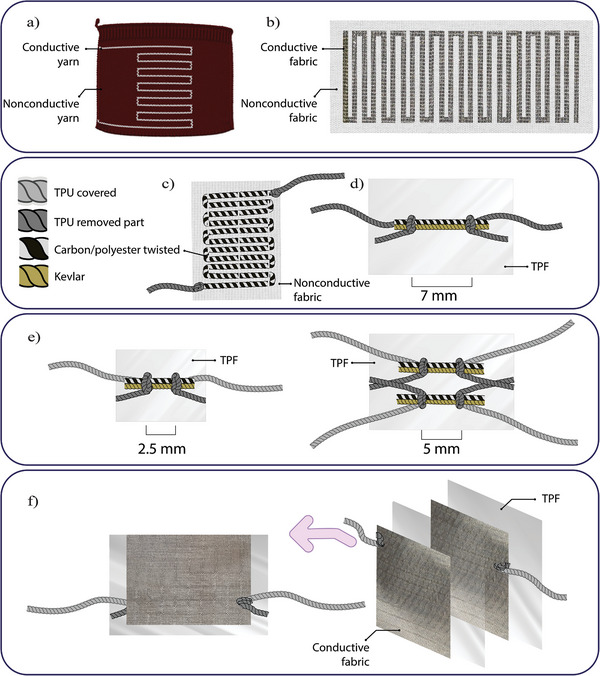
The early‐produced textile‐based RC filtering samples: a) sample R_Knit_: 2 MΩ. b) sample R_Fabric_: 5 kΩ. c) sample R_CFY1_: 66 MΩ. d) sample R_CFY2_: 2.5 MΩ. e) sample R_CFY3_: 1 MΩ, (left) single line design and (right) parallel connection design. f) Sample C_B‐RS2_: 1 nF, (left) top view and (right) decomposed view.

In the initial development stage, five different resistor samples were created using knitting, laser cutting, stitching, and knotting techniques. The details of the manufacturing processes are provided in Section S1 (Supporting Information). They are summarized below as follows:

- Knitting‐based resistor (R_Knit_): Created by knitting stainless steel fiber yarn on a knitting machine. This sample achieved a resistance of 2 MΩ, however, the value was unstable due to the relaxation of loop structures. See **Figure** **2**a). The burgundy material represents the non‐conductive knitted base, onto which the silver‐colored conductive yarn was knitted to create the resistive path.
- Conductive‐fabric‐based resistor (R_Fabric_): Fabricated from conductive fabric made of 100% polyamide with a silver coating to prevent loop relaxation. This sample achieved 5 kΩ which is a relatively low value, requiring the capacitance to be in the hundreds of nF range. Furthermore, its dimensions were large, measuring 27 × 7.5 cm^2^. See Figure 2b). The white material represents the non‐conductive base fabric, with the silver‐colored conductive fabric adhered on top to create the resistive path.
- Carbon‐yarn‐based resistor (R_CFY1_): Made by hand‐sewing carbon filament yarn. This sample achieved a resistance of 66 MΩ. See Figure 2c). The white material represents the non‐conductive base fabric, with the resistive path stitched on top. The black (carbon) and white (polyester) twisted yarn represent the resistive path.
- Carbon‐yarn‐based resistor (R_CFY2_): Produced by hand‐knotting a single line of carbon filament yarn. This resistor achieved a resistance of 2.5 MΩ. See Figure 2d. The black (carbon) and white (polyester) twisted yarn represent the resistive part, while the yellow yarn (Kevlar) provides structural support. Both are encapsulated in a transparent thermoplastic film, with the gray colored thermoplastic polyurethane (TPU)‐covered conductive yarn providing the electrical connection.
- Carbon‐yarn‐based resistor (R_CFY3_): Made by hand‐knotting two parallel lines of carbon filament yarn. This design achieved a resistance of 1 MΩ. See Figure 2e). The black (carbon) and white (polyester) twisted yarn represent the resistive path. Two alternative designs for a single‐line (left) and parallel‐line (right) resistors are shown.

The primary design goal for the final sample was to create small‐sized resistors with sufficiently high resistance values. For the main resistor, carbon filament twisted with polyester yarn (CFY) was selected due to its high resistance (400 ± 40 MΩ m^−1^). The carbon filament preparation process involved unraveling the CFY twist, isolating the carbon filament part, which was 40 cm in length. The filament was then folded 8 times, reducing its size to 5 cm, and hand‐twisted at each fold.

**Figure 3:**
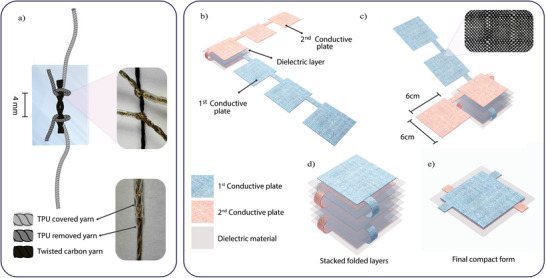
The produced textile‐based filtering samples' design. a) Resistor sample R_CFY4_ (90 kΩ). b) Construction of the C_B‐RS3_ sample by folding the cut squares of the conductive fabric and interleaving with dielectric layers (materials and layers are color‐coded for visual comprehension). c) C_B‐RS3_ sample after folding halfway through. d) The full stack of folded fabric and dielectric material of C_B‐RS3_ sample. e) The final form of Capacitor sample C_B‐RS3_ (14 nF).

To create conductive transmission lines for easy circuit connection at both ends of the thin carbon yarn, Shieldex silver‐coated 235/36 dtex transparent thermoplastic polyurethane (TPU) coverd yarn was used.his TPU‐covered conductive yarn was selected for its shielding properties, which protect the silver coating from external factors while electrically isolating the transmission line. To facilitate circuit connections, 3 cm of TPU was removed from both ends of the TPU‐covered yarn. Two TPU‐removed conductive yarns, each separated by 5 mm, were knotted onto the twisted carbon yarn. The resistor components were then positioned between two 100‐micron ThermoPlastic Film (TPF) layers, and heat and pressure were applied at 80○C for 7 s. The TPF encapsulation provides effective protection of internal components while maintaining the device's lightweight profile. The film's durability ensures the resistor can withstand everyday wear without compromising its mechanical integrity. The final sample, labeled R_CFY4_, was a compact 90 kΩ resistor with dimensions of (2 × 3 cm^2^) See **Figure** **3**a) for design illustrations. The figure shows the black twisted carbon yarn of the resistor, with Shieldex silver‐coated yarn knots at both ends (after removing TPU at the edges for electrical conduction). The entire structure is encapsulated by the transparent TPF, and the TPU‐covered yarn facilitates isolated electrical connection.

Regarding the production of capacitors, three different samples were initially fabricated using folding and pressing methods. The details of their production are discussed in the Section S1 (Supporting Information). They are summarized as follows:

- Folding Technic‐tex fabric‐based capacitor (C_T‐tex_): Made by folding Shieldex Technic‐tex P130 conductive fabric and FL120VS Polyethylene vacuum nylon layers. This sample achieved 7 nF but was bulky and unwearable, measuring 5.5 × 5.5 × 1.5 cm^3^.
- Folding large Bremen RS fabric‐based capacitor (C_B‐RS1_): Fabricated by folding a large section of Shieldex Bremen RS, a metalized woven fabric with pure silver, along with layers of thermoplastic film. It achieved 33 nF but was still large, measuring of 12 × 8 × 0.25 cm^3^.
- Folding small Bremen RS fabric‐based capacitor (C_B‐RS2_): Made by folding a small section of Shieldex Bremen RS, metalized with pure silver and combined with thermoplastic film layers. This sample achieved 1 nF and was designed to complement resistors in the range of 1 MΩ. See Figure 2f. The transparent film represents the dielectric layer, and the textured silver material acts as the conductive fabric capacitance electrodes.

The primary objective of the final capacitor design was to select a capacitance value that would appropriately complement the resistor to achieve the desired cutoff frequency for the low‐frequency filter. In addition to this, the capacitor needed to be compact enough for seamless integration into wearable garments. For this reason, Shieldex Bremen RS, a woven conductive fabric, was chosen as the capacitor's electrodes material. Woven fabrics were preferred over knitted fabrics due to their greater durability and reduced elasticity, which are advantageous for wearable applications. The conductive fabric electrode areas were precisely cut to dimensions of 6 × 6 cm^2^ using a laser cutting machine.

Inspired by the folding method used by Terada et al.,^[^ ^36^ ^]^ the capacitor plates were cut such that four identical pieces (representing one plate of the capacitor) were linked by a section of conductive fabric. To maximize the capacitance value, a thinner dielectric material–8‐micron Linear Low‐Density Polyethylene (LLDPE) stretch film–was selected, offering improved wearability compared to previous, thicker designs. The plates of the capacitor were positioned perpendicular to each other, with a 7.5 × 7.5 cm^2^ piece of LLDPE film placed between them before each fold. The layers were bonded together using 3M Vernis isolant transparent 81 042 adhesive spray, ensuring no air bubbles were trapped between the layers. A heat press was then used to compress the layers at 30°C for 10 s. This process was repeated for each fold to ensure a strong bond and consistent dielectric performance.

To protect the sensitive dielectric material and enhance the durability of the capacitor, both sides of the assembled structure were covered with 8 cm^2^ of TPF using a heat press at 80°C for 10 s. This final step resulted in a capacitor with a capacitance of 14 nF. The design of the completed capacitor (C_B‐RS3_) is illustrated in Figure 3b–e. The folding steps are started by laying the two strips of conductive fabric cut into four connected squares (the capacitor's conductive plates) at a 90°from each other. A piece of cut LLDPE film is placed on the first square of one plate at the angle and the other plate's first square is laid on top of it, LLDPE film piece is placed again on top of the structure and the second square of the first plate is folded on top of it. The process is repeated until all squares are folded and interleaved with LLDPE film pieces and the final compact form is constructed.

### 3.2 Evaluation of Textile‐Based Samples as Acceptable Filter Candidates

To evaluate the effectiveness of the samples produced in Section 3.1 as wearable filters, three key criteria were selected for testing in this study: the frequency response of the filter, the effect of skin proximity, and the impact of skin contact impedance.

#### 3.2.1 Evaluation of Frequency Response of the RC Circuit

In this study, we are particularly interested in the magnitude of the frequency response, as the phase response is not critical for noise reduction in ECG applications.^[^ ^35^ ^]^ The frequency response is measured using a function generator and a two‐channel oscilloscope to produce the magnitude Bode plot. For this purpose, the National Instruments VirtualBench, which integrates both a generator and an oscilloscope, was employed. The measurement setup was configured to sample twenty points per decade, with each point measured twenty times and then averaged to yield the final data for the plot. The excitation signal is a 10 V peak‐to‐peak (Vpp) sinusoid, and the frequency sweep covers the range from 1 Hz to 1 MHz.

To pinpoint the effect of the textile components accurately, they are first tested individually on the bench. This is done by connecting textile resistors to standard electronic capacitors in low pass filter configuration and the other way around for textile capacitors. The best‐performing samples are identified by denoting the theoretical cutoff frequency for each filter and monitoring the behavior of the frequency response. Using those samples a full textile‐based RC filter is then created and tested by comparing the shape of the response curve to a simulation created via LTspice.

**Figure 4:**
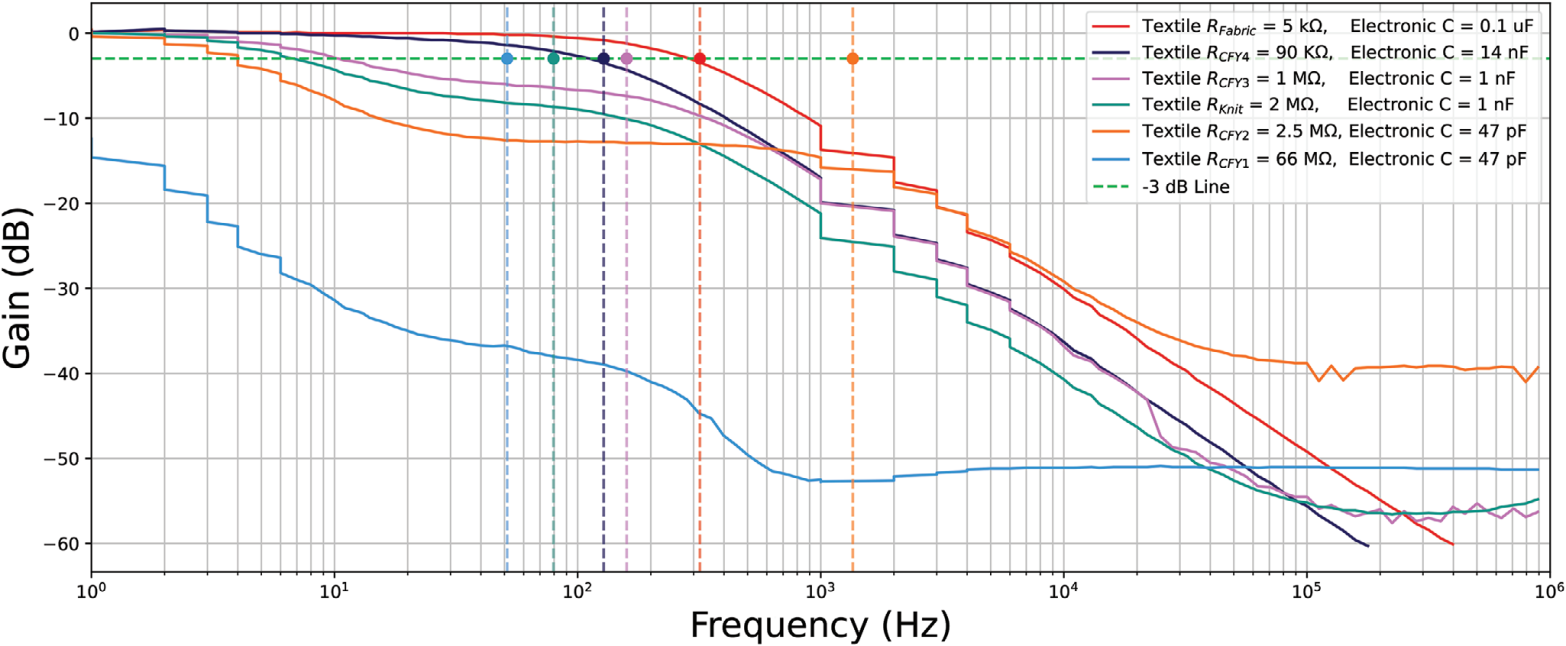
Frequency response of the textile‐based R samples.

The frequency responses of the RC filters, each using different textile‐based resistors, are shown in **Figure** **4**. The colored dots in the graph represent the ideal cutoff frequency for each filter, where the response curve should ideally cross the –3 dB line. The textile‐based resistor values varied depending on the material and construction method used. Capacitors were selected from available components in the lab to ensure the resulting cutoff frequency remained within the low‐frequency range for visual consistency. From the results, it is clear that the smaller the resistor value, the better the filter's performance. Specifically, the gain remains stable before the cutoff frequency, and the roll‐off starts smoothly after it. Conversely, as the resistor value increases, the performance deteriorates, with the gain dropping earlier than expected and the roll‐off occurring in a non‐uniform manner. However, in terms of wearability, the sample R_Fabric_ (5 kΩ), which displayed the best response curve, required a large surface area (27 × 7.5 cm^2^) due to the highly conductive material used. Thus, the sample R_CFY4_ (90 kΩ) was chosen as the optimal option, offering a balance between good performance and compact size (2 × 3 cm^2^).

**Figure 5:**
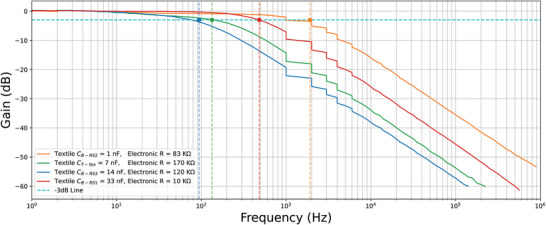
Frequency response of the textile‐based C samples.

The results of the next experiment, where textile capacitors were connected to standard electronic resistors, are shown in **Figure** **5**. From these results, it is evident that changes in capacitor values, in the range of a few nanofarads (nF), do not significantly affect the quality of the frequency response curve. However, in terms of wearability, sample C_T‐tex_ proved too bulky (5.5 × 5.5 × 1.5 cm^3^) to be suitable for embedding in clothing. Sample C_B‐RS1_ also covered a large surface area (12 × 8 × 0.25 cm^3^), which made it less ideal for wearable applications. On the other hand, samples C_B‐RS2_ and C_B‐RS3_ were compact enough to be embedded in an electrode, with sample C_B‐RS3_ having a higher capacitance value (14 nF) compared to sample C_B‐RS2_ (1 nF). By combining the results from Figures 4 and 5 and balancing performance with wearability, the final design for the RC filter was selected: a resistor with a value of 90 kΩ and a capacitor with a capacitance of 14 nF, resulting in a cutoff frequency close to 100 Hz (approximately 126 Hz).

#### 3.2.2 Evaluation of the Effect of Skin Proximity and Skin Impedance

**Figure 6:**
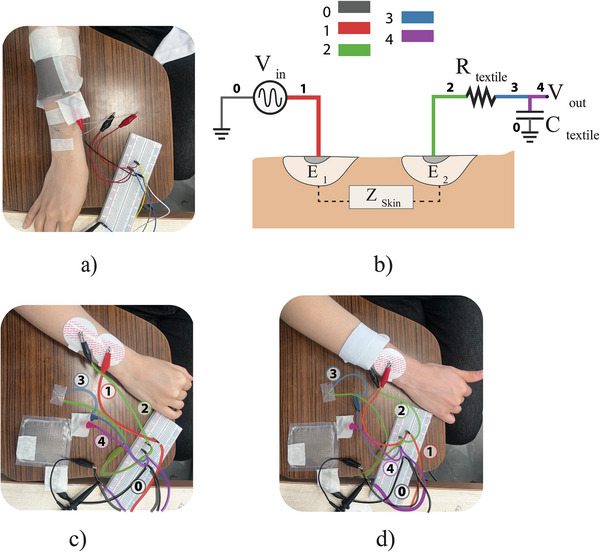
Experimental setup for assessing the effect of skin proximity and skin impedance experimental setup. a) Measuring frequency response of the samples against skin. b) Measuring the effect of the skin contact impedance on the frequency response of the filter by connecting the textile‐based R and C to c) textile‐based electrode and d) wet electrode.

It is well known that skin proximity and contact with the conductive capacitive plates can alter the capacitance due to parasitic effects. Therefore, after identifying the optimal textile‐based RC configuration, the components are isolated by encasing them in TPF (thermoplastic film). These encapsulated components are then pressed and taped against a subject's skin, as shown in **Figure** **6**a, and the frequency sweep is performed again. The resulting frequency response is compared to the benchtop test data to assess any variation in performance.

In the majority of previous work, the attempts at filtering the signals and observing the quality of the output signal have been focused on offsetting the skin‐to‐electrode contact impedance and then achieving a filtering effect. Furthermore, there had not been a report of frequency response done for textile‐based filter performance that involves the effect of skin‐to‐electrode contact impedance. This is rather understood, as the frequency sweep requires control over the excitation signal, while most studies focus on the ECG signal as the focal point of application and measure the final quality of the captured signal. Here, the concern is that the impedance on the contact point will offset the value of the resistor, which is to be connected in series with the electrode in a manner that will result in a major shift of the cutoff frequency. If the case is as such, then adjustment of the resistor or capacitor values would be needed to compensate for the shift and bring the frequency curve back to a preferable place.

To address this, we conducted an experiment to measure the frequency response of the filter through the subject's skin. The experimental setup is illustrated in Figure 6b. The excitation signal is applied to the subject's skin via gel electrode E_1_, while a second electrode, E_2_, is placed 2 cm away and connected to the textile‐based filter. In this configuration, the excitation signal is assumed to pass from E_1_ to E_2_ through the skin, encountering the contact impedance on its path to the filter circuit. The resulting frequency response curve is compared to benchtop results to ensure no significant deviation in the cutoff frequency. If any discrepancies are observed, adjustments to the component values would be in order.

Two types of electrodes were tested at the contact point (E_2_) and compared. The first was a standard wet Ag/AgCl electrode, and the second was a dry textile‐based electrode. This comparison allows for the evaluation of the textile electrode's performance relative to the wet electrode. For this purpose, we designed a portable textile‐based electrode by cutting 5 × 5 cm^2^ rectangular pieces from Shieldex Technic‐tex conductive fabric. These pieces were then sewn onto a 6 × 11 cm^2^ woven fabric, with the wrong sides facing each other and one edge left open for turning the fabric inside out. To achieve a curved structure that enhances adherence and contact pressure, a spacer material was incorporated. After filling the electrode with the spacer material, the open edge was stitched closed. The experimental setup for both electrodes is shown in Figure 6c,d. The connection wires are color‐coded to match their respective connections in the schematic of Figure 6c, ensuring visual coherence.

**Figure 7:**
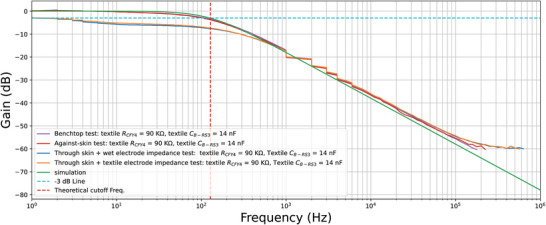
Frequency response of the skin and contact evaluation experiments.

The frequency response results from the skin contact and impedance tests, comparing the wet electrode and dry textile‐based electrode, are presented in **Figure** **7**. The frequency response curve of the benchtop test closely matches that of the simulation. The frequency response of the skin proximity effect also mirrors the simulation, indicating that the isolation provided by TPF encapsulation is adequate to prevent parasitic drift effects. Similarly, the curves obtained from the skin impedance test setup show a slight reduction in gain levels in the passband compared to the simulation, but the overall shape of the response curve remains intact. Notably, no major shift in the cutoff frequency or the rolloff shape was observed for either the wet or dry textile‐based electrodes. These results suggest that no adjustments to the component values are necessary.

### 3.3 Evaluation of Samples Under Repeated Wear

The proposed textile‐based filters are designed to be integrated into clothing, specifically within ECG electrodes. These filters will undergo continuous bending due to the garment's response to the wearer's body contours during movement. Therefore, it is crucial to evaluate the durability of the resistive and capacitive textile components under continuous bending to ensure that their properties do not degrade significantly over time. In an independent study, Li et al.^[^ ^27^ ^]^ developed a novel testing method to assess the sensing capabilities of knitted strain sensors using a 3D curved surface. Inspired by their work, we designed a 3D bending test to simulate the long‐term wearability of the produced samples, which would replicate the continuous bending and pressing of the body contour onto the samples.

**Figure 8:**
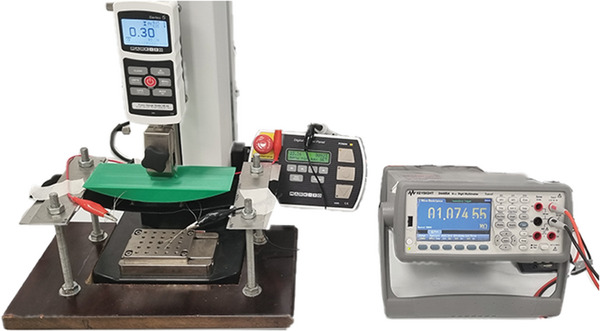
Bending test set up.

The extent of bending that an ordinary t‐shirt experiences on the body was determined by using a 30 cm flexible rod placed across the participant's body, with the deepest part of the bend measured to be 2.5 cm. Consequently, the 3D curved head used to bend the samples was fabricated via 3D printing with dimensions of 30 cm in length, 7 cm in width, and a maximum depth of 2.5 cm. This head was attached to the upper grip of the Mark‐10 tensile testing machine. To ensure that the sample could be bent freely, it was suspended between two handles. Specifically, the sample under test was sewn onto a knitted piece of jersey fabric, and the fabric was clamp‐suspended, allowing it to be pressed vertically. The experimental setup is shown in **Figure** **8**.On the left, the tensile machine stands with the designed green curved head pressing on the sample, which is stitched to the base fabric suspended from the metallic side clamps. On the right, the multimeter continuously records the sample's values.

The tensile testing machine was configured with a speed of 500 mm min^−1^, a travel distance of 25 mm, and 100 cycles of repetition. The sample under test was connected to a Keysight 34465A digital multimeter, which continuously measured its value. A program running on a PC was used to track and collect distance data from the machine, as well as the measured values from the multimeter, throughout the test.

The tolerance was calculated from the bending test results using the following formula:

(2) $$tolerance=\frac{x_{max}-x_{min}}{x_{min}}\cdot 100\%,$$

where *x*_*max*_ and *x*_*min*_ represent the maximum and minimum values measured during the test, respectively.

**Figure 9:**
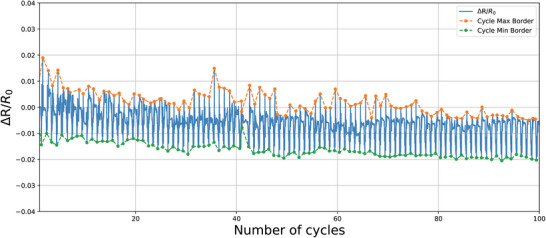
Resistance trend in bending test with resistor sample 1 over 100 cycles.

**Figure 10:**
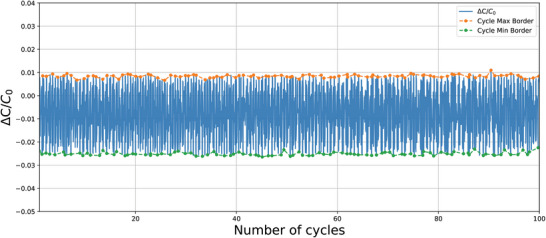
Capacitance trend in bending test with capacitor sample 1 over 100 cycles.

For this test, two resistor samples (R sample R_CFY4_ from Section 3.1) and two capacitor samples (C sample C_B‐RS3_ from Section 3.1) were produced and tested accordingly. The tolerance results are presented in **Table** **1**, while **Figures** **9** and **10** illustrate the variation in resistance and capacitance for resistor sample 1 and capacitor sample 1, respectively, over 100 cycles of bending. $R_{0}$ represents the initial measured resistance, and Δ*R* is the difference between the initial resistance and the instantaneous resistance measured throughout the test. The rate of change is calculated by taking the ratio of these values. The maximum and minimum measured rates of change in resistance and capacitance per cycle are highlighted with orange and green dots, respectively. The general trend for the maximum and minimum rates of change is tracked over all cycles with dashed lines in the corresponding colors.

**Table 1:** Tolerance results for bending test samples.

Type	Tolerance of	Tolerance of	Average
	sample 1 [%]	sample 2 [%]	tolerance [%]
Resistor	4.03	3.70	3.86 ± 0.16
Capacitor	2.13	3.84	2.99 ± 0.85

The results of the bending test show that the textile‐based resistors achieved an average tolerance of 3.86%, while the textile‐based capacitors exhibited an average tolerance of 2.99%. These values fall within the typical commercial tolerance range for standard resistors and capacitors, which is generally reported as 5–10%. Following the integration of the filters into clothes, additional bending tests were performed directly on the body of the subject wearing the integrated garment while performing different activities, the details of the test can be found in Supplementary Information Section 3.

### 3.4 Evaluation of the Textile‐based RC Filters in ECG Noise Reduction

Following the evaluation of the efficiency of the produced textile components, the next phase of the study focuses on assessing the performance of the textile‐based filters in reducing noise within an ECG application. This evaluation consists of two procedures: the first involves the integration of the filter into clothing, while the second consists of real‐world data collection and subsequent signal processing.

#### 3.4.1 Integration of Textile‐based Electrodes and Filters Into Clothes

To integrate the proposed RC filters into a wearable system, a smart t‐shirt incorporating e‐textiles was developed. The t‐shirt was fabricated using seamless manufacturing techniques to embed textile electrodes. The fabric was composed of 93% polyamide and 7% elastane for the main yarn, while the conductive yarn consisted of Shieldex 78/18 dtex +B silver‐plated fibers. A three‐electrode ECG measurement system, incorporating textile‐based electrodes, was seamlessly integrated using a Santoni 15‐inch diameter circular knitting machine.

**Figure 11:**
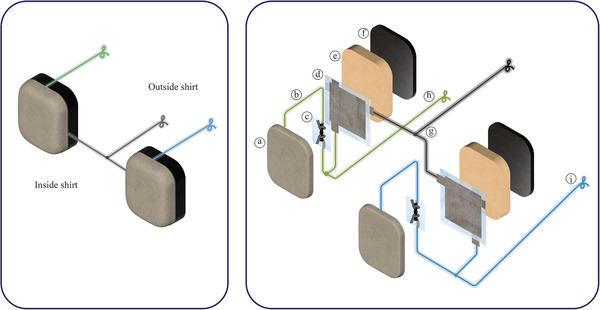
Integration of textile‐based filters. (Left): Compact view of electrodes. (Right): Decomposed view of electrodes. a) Conductive fabric. b) TPU‐covered conductive yarn. c) Textile‐based resistor. d) Textile‐based capacitor. e) Sponge. f) T‐shirt material. g) Connection line to the outside of the t‐shirt (Ground). h) Connection line to the outside of shirt (Amplifier positive input). i) Connection Line to the outside of the t‐shirt (Amplifier negative input).

The electrodes were positioned on the left front side of the t‐shirt, with two electrodes surrounding the heart area, while the third, serving as the reference grounding electrode, was located below them in the abdominal region. The t‐shirt design featured a two‐layer construction, with the conductive yarn forming the electrodes embedded in the inner layer, which directly contacts the skin. A sponge layer was inserted between the inner and outer layers to apply the necessary pressure to the electrodes in contact with the body. The resistive and capacitive filtering components were placed between the conductive electrode surface and the sponge, as shown in **Figure** **11**. Two filters were integrated, one for each of the electrodes surrounding the heart, while the third electrode, which serves as the ground, remained unfiltered.

To connect the resistor to the electrode, TPU was removed from the end of one of the resistor's transmission lines to expose the underlying conductive yarn, which was then knotted to the electrode using a crochet hook. The second transmission line of the resistor was manually knotted to one of the transmission lines of the capacitor after removing the TPU from both edges. A common line was then drawn from this junction point to the outside of the t‐shirt. This process was repeated for the other electrode. The remaining loose ends of the capacitor's second transmission lines were tied together at a junction point, and their common line was routed to the outside of the t‐shirt as a grounding pint.

**Figure 12:**
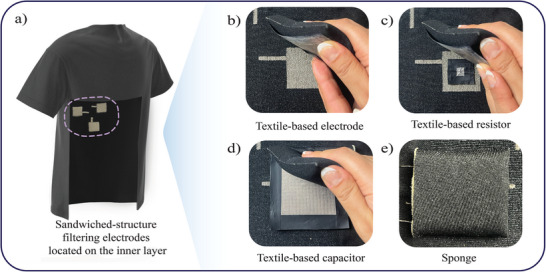
The integrated filtering components inside the t‐shirt. a) The designed t‐shirt with a cut made to show the electrodes inside. b) The back of the textile electrode. c) The textile‐based resistor laid on the electrode. d) The textile‐based capacitor laid on top of the resistor, and e) The sponge that will provide a cushioning effect to the electrode stitched on top of the components.

The resulting junction points between transmission lines were sealed with heat‐pressed TPF to ensure electrical isolation. Finally, the components were arranged flat against each other, with the sponge placed on top. The sponge was hand‐stitched to the t‐shirt material, while the transmission lines routed to the outside of the t‐shirt were gathered and stitched along the outer surface, creating contact points for crocodile clips. Additionally, a direct transmission line was established from the conductive face of the electrode (bypassing the filtering path) to the outside of the t‐shirt, enabling a comparison between signals captured with and without the filters. **Figure** **12** shows the layers of the integrated components with a) showing the t‐shirt with hypothetical cut peering into the electrodes embedded inside, b) showing the back of the conductive electrode, c) showing the resistor laid onto the electrode, d) showing the capacitor laid on top of the resistor, and e) showing the sponge layer to be stitched covering all components below. In the illustration, the back of the t‐shirt is cut to show the location of the electrodes on the inner layer.

#### 3.4.2 Noise reduction performance of the textile‐based RC filter

**Figure 13:**
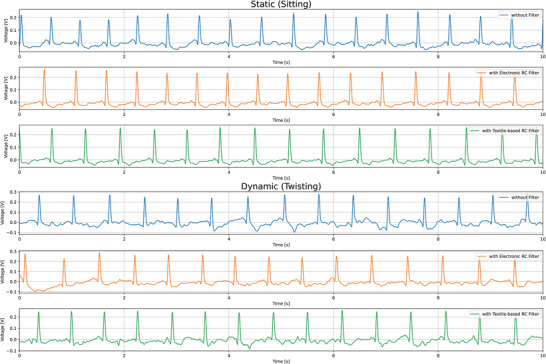
ECG signals captured during sitting (above) and during twisting of the upper torso (below).

**Figure 14:**
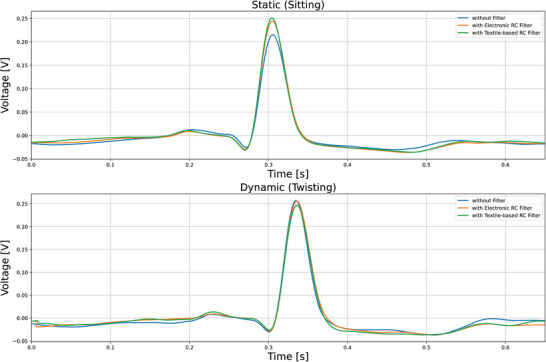
Average ECG beat templates during sitting (above) and during twisting of the upper torso (below).

To evaluate the performance of the designed filters, first, the efficacy of textile electrodes in measuring ECG signals was established. The details of their performance in comparison to Ag/AgCl gel electrodes can be found in Supplementary Information Section 2. Following that, three different wear cases were tested. The first case involved measuring the ECG signal directly from the textile electrodes without any filtering. The second case utilized standard electronic filters, while the third case employed textile‐based filters embedded within the electrodes to measure the ECG signal. Two types of activities were tested for each of the three cases: a static activity (sitting) and a dynamic activity (twisting). **Figure** **13** illustrates the ECG signals recorded for each of these cases. From the figure, it is evident that fluctuations in the signal baseline (between the peaks) are more prominent in the case without filters, whereas the cases using electronic and textile‐based filters show improved signal stability. **Figure** **14** presents the average ECG beat template. It is apparent from the figure that the uniformity of the average beat shape and the amplitude of the peaks are similar in both the electronic and textile‐based filtering cases, whereas they differ notably from the case with no filtering.

The calculated signal‐to‐noise ratio (SNR) results for the experiments are summarized in **Table** **2**. The SNR values clearly demonstrate that both the electronic and textile filters enhance the SNR of the ECG signals compared to the case where no filter is used, which aligns with the visual assessment in Figure 13. Furthermore, the SNR values for the electronic and textile filters are sufficiently close, suggesting that textile‐based filters perform similarly to standard rigid electronic filters.

**Table 2:** SNR results for ECG measurement experiments.

Test	without Filter	with Electronic	with Textile‐based
		RC Filter [dB]	RC Filter [dB]
Static (Sitting)	14.74	23.67	25.25
Dynamic (Twisting)	7.76	11.85	11.51

## 4 Conclusion

In this study, we propose a novel design for a full textile‐based low‐frequency RC filter aimed at ECG noise reduction. To achieve this, various textile materials and methods were explored to identify the most suitable samples for the filter design. The samples were evaluated and compared based on their filtering frequency response, leading to the identification of the optimal design approach. Furthermore, the performance of the textile‐based filter was assessed under conditions relevant to wearability, including the effects of skin isolation and contact impedance–key factors in biophysical signal monitoring. The results demonstrate that the filtering performance is well‐preserved under these wearability conditions.

In addition, the tolerance of the textile‐based filtering components under wearability strain was evaluated through a custom‐designed bending test. The results indicate that the tolerance of the samples falls within the standard commercial tolerance range for electronic components. To assess the practical performance of the filter, it was integrated into an ECG monitoring application using textile‐based electrodes embedded within a t‐shirt. The measured signal‐to‐noise ratios (SNRs) show that the textile‐based filters effectively suppress ECG signal noise, performing on par with standard electronic filters when compared to capturing the signal without any low‐pass filter.

Looking ahead, the development of an embedded filter within a single material layer will be pursued, enabling the creation of a compact, unified filter unit. Additionally, further miniaturization of the filtering components will be explored to facilitate the development of cascaded higher‐order filters. Lastly, the potential for creating tunable cutoff frequency filters, leveraging the stretchable properties of textile materials, will be investigated.

## 5 Experimental Section

**Figure 15:**
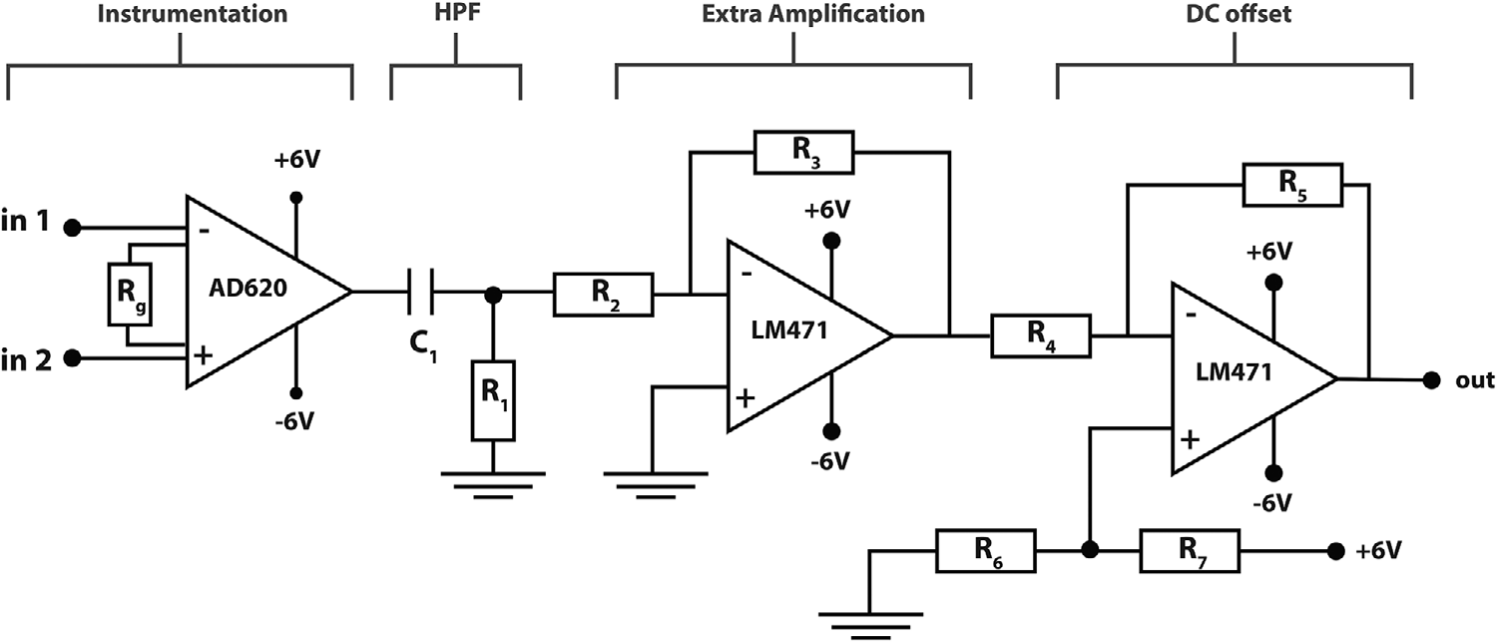
ECG conditioning circuit. (R_g_: 1 kΩ, C_1_: 1000 µF, R_1_: 5.8 kΩ, R_2_: 1 kΩ, R_3_: 91 kΩ, R_4_: 12 kΩ, R_5_: 1 kΩ, R_6_: 22 kΩ, R_7_: 91 kΩ).

The ECG signal was captured using the AD920 (Analog Devices) instrumentation amplifier, laid out in the designed conditioning circuit shown in **Figure** **15**. The measurement system employs a three‐electrode configuration. The setup consists of two main electrodes surrounding the heart, where the textile‐based filter is embedded, and connected to the inputs of the instrumentation amplifier. The third electrode, located on the abdominal area, is connected to the common ground of the circuit. The circuit was designed with the instrumentation amplifier with gain of 50 as the first stage to which the electrodes connect to. A high‐pass filter with a cutoff frequency of 0.02 Hz was added in the subsequent stage. The signal was then inverted using a negative feedback operational amplifier (Op‐amp) with a gain of 1, preparing it for the final stage. In this stage, the signal undergoes a DC shift of 1 Volt and is inverted once more via the final Op‐amp. This voltage shift ensures that the ECG signal remains within the range of the Analog‐to‐Digital Converter (ADC) in the subsequent sampling stage, preventing any negative levels.

The output of the circuit was connected to the 12‐bit ADC of the STM32WB55 microcontroller with a measurement range of 0–3.3V, and a sampling rate of 2 kHz. The sampled data were transmitted to a PC via a serial connection at a baud rate of 115 200. On the PC side, a Python program was used to receive and save the transmitted data. Once the data was captured, a digital notch filter at 50 Hz was applied to remove power line noise, followed by a high‐pass filter at 0.5 Hz to eliminate electrode baseline wandering effects. No low‐pass filter was applied to allow for the observation of the effects of the designed filter.

The quality of the captured ECG signal was assessed using the Signal‐to‐Noise Ratio (SNR) metric. Since ECG signals are subject‐specific and vary based on measurement location, the SNR was calculated using the template matching method, defined as:

(3) $$SNR\left[dB\right]=10\cdot log_{10}\left(\frac{P_{signal}}{P_{noise}}\right),$$

where *P*_*signal*_ and *P*_*noise*_ represent the pure signal power and the noise power, respectively. The pure signal was estimated by averaging the filtered and segmented heartbeats centered around the R‐peak, which were used to create a reference beat template. The noise was then estimated by subtracting the reference beat from the captured heartbeat, and the SNR was calculated. This process was repeated for all recorded beats, and the results were averaged.

To evaluate the effect of the designed filter, data was recorded for three different cases: the first case involved no filters connected to the instrumentation amplifier, the second used standard electronic filters (R = 0.7 MΩ, C = 4.7 nF) onnected to the amplifier's input, and the third case incorporated the textile‐based filters. A single adult female subject, who consented to the experiment, wore the t‐shirt during testing. Two types of activities were recorded: a static test in which the subject remained seated with her back straight, and a dynamic test in which the subject twisted her upper torso left and right while seated. Proper circuit connections were made before each test (with or without the electronic or textile‐based filters).

In the first experiment, the direct transmission lines from the electrodes were connected to the amplifier input via crocodile clips, without any filter. The subject performed the static test first, with data recorded for 10 s, followed by the dynamic twisting test, with another 10 s of data recording. The process was repeated for the second case with standard electronic filters, which were connected to the amplifier input and the direct electrode transmission line from the previous test. Finally, the third case was conducted by disconnecting the electronic filters and connecting the textile‐based filters, integrated into the electrodes, to the amplifier input via the transmission lines extending outside the t‐shirt. The same sequence of tests was repeated for this case.

## Conflict of Interest

The authors declare no conflict of interest.

## Author Contributions

N. A. contributed to conceptualization and study design, methodology, experiment design, instrumentation and experimental setup preparation, filter integration design, experimentation, data collection, formal analysis, visulization, drafting the original manuscript, and editing. I. Y. was responsible for designing and producing textile‐based samples, designing the bending test, setting up the bending test and collecting data, filter integration, visualization, drafting the original manuscript, and editing. K. O. handled the instrumentation of the bending test, as well as reviewing and editing. M. S. C. worked on the design and production of the knitted textile‐based resistor sample and filter integration. A. T. A. contributed to conceptualization, methodology, supervision, and validation. O. A. was involved in project administration, conceptualization, methodology, supervision, and validation. G. I. participated in project administration, conceptualization, methodology, supervision, validation, reviewing, and editing.

## Supporting information

Supporting Information

## Data Availability

The data that support the findings of this study are available from the corresponding author upon reasonable request.
