# Supplementary material for: Textile‐based Low‐frequency RC Filter for Noise Reduction in ECG signals
Source: Glob Chall. 2025 Feb 11;9(3):2400237. doi: 10.1002/gch2.202400237 (PMC11891578; doi:10.1002/gch2.202400237)
Supplement: Supplementary file 1 — Supporting Information [file GCH2-9-2400237-s001.pdf]

Global Challenges
Open Access

Supporting Information

for *Global Challenges.*, DOI 10.1002/gch2.202400237

Textile-based Low-frequency RC Filter for Noise Reduction in ECG signals

*Nada Al-azzawi, Irem Yunculer, Kadir Ozlem, Munire Sibel Cetin, Asli Tuncay Atalay, Ozgur Atalay and Gökhan Ince**

Section 1: Production of textile based resistors and capacitors

Table 1: Summary of the produced resistors samples methods and materials.

	**Production method**	**Materials**	**Material measured resistance**	**Machines**	**Value**
**Sample R_{Knit}**	Weft knitting	-Resistance yarn: Bekintex Fibres BK 50/1, 80% polyester and 20% stainless steel fibers.<br><br>-structure yarn: Karan Textile Ne 24/2 cotton yarn.	Unstable	Knitting: Shima Seiki NSVR-122 14-gauge computerized flat-bed knitting machine.	2 MΩ (unstable)
**Sample R_{Fabric}**	Laser cutting and adhesion by heat press	-Conductive fabric: Shieldex Tulle 100% polyamide with a silver coating.<br><br>-base material: Voile woven fabric 60/1.<br><br>-adhesive film for other layers: 100 micron ThermoPlastic Film (TPF).	<1.4 Ω/□	-for cutting conductive fabric: Universal laser systems VLS cutting machine<br>-to apply heat and pressure: Heat Press Model 1702	5 KΩ
**Sample R_{CFY1}**	Hand sewing	-Resistance yarn: Beşhan Textile Carbon Filament twisted with polyester Yarn (CFY).	400 MΩ/m ± 40 MΩ/m	N/A	66MΩ
**Sample R_{CFY2}**	Creating knots by hand and fusing with TPF	-Resistance yarn: Beşhan Textile CFY.<br><br>-structure support yarn: Dupont Kevlar yarn (Ne16/2).<br><br>-transmission line: Shieldex	400 MΩ/m ± 40 MΩ/m	to apply heat and pressure: Heat Press Model 1702	2.5 MΩ

		235/36 dtex, TPU-covered yarn.			
**Sample R_{CFY3}**	Creating knots and fusing with TPF	-Resistance yarn: Beşhan Textile CFY.<br><br>-structure support yarn:<br>Dupont Kevlar yarn (Ne16/2).<br><br>-transmission line: Shieldex 235/36 dtex, TPU-covered yarn.<br>-adhesive film for other layers: 100-micron TPF.	400 MΩ/m ± 40 MΩ/m	to apply heat and pressure: Heat Press Model 1702	1 MΩ
**Sample R_{CFY4}**	Creating knots and fusing with TPF	-Resistance yarn: Beşhan Textile CFY.<br><br>-transmission line: Shieldex<br>235/36 dtex, TPU coverd yarn.<br><br>-adhesive film for other layers:<br>100 micron TPF.	400 MΩ/m ± 40 MΩ/m	to apply heat and pressure: Heat Press Model 1702	90 KΩ

Table 1 summarizes the materials and methods used to produce resistor samples.

To produce resistor sample R_{Knit}, the knit structure was designed using APEX4 software, and the sample was created on a Shima Seiki flat-bed knitting machine. A steel-based yarn (BK 50/1) was employed to create conductive loops while a cotton yarn (Ne 24/2) was utilized for the main structure of the knitted sample. Since the thickness of the conductive yarn is not suitable for our 14-gauge knitting machine, it is simultaneously fed with the cotton yarn to the machine. The conductive yarn was embedded into a jersey fabric structure in a series of single loops, as shown in Figure 1 a). Due to the resistance value of the conductive yarn used being unstable, the resultant resistive sample had a fluctuating resistance of around 2 MΩ. This fluctuation was further amplified due to the nature of knit structures being that of loops and stretchable, rendering the resistance value non-constant as the contact resistance between loops alters when the fabric stretches.

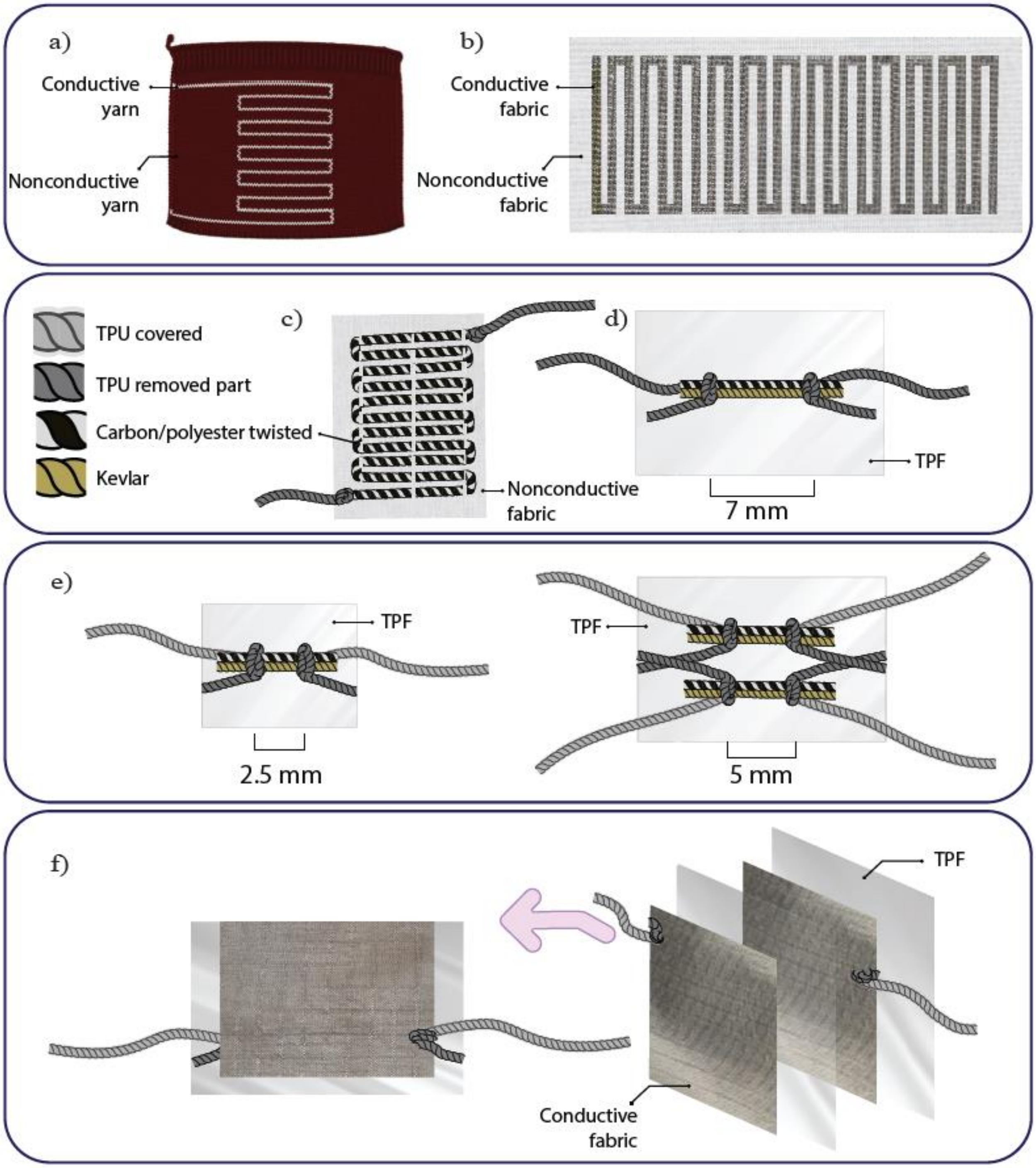

Figure.1 The early-produced textile-based RC filtering samples: a) Sample R_{Knit}. b) Sample R_{Fabric}. c) Sample R_{CFY1}. d) Sample R_{CFY2}. e) Sample R_{CFY3} (left) single line design and (right) parallel connection design. f) Sample $C_{B\text{-}RS3}$. (left) top view and (right) decomposed view.

To counteract the issue of unstable resistance, the production of sample R_{Fabric} involved the usage of two types of fabric, a conductive one for the resistance part and a woven fabric for structure support. A structure of a long-winded track as shown in Figure 1 b) with a width of 0.2 cm and a total length of 533 cm was created using Coral Draw software. Before cutting the knitted conductive Shieldex Tulle fabric, it was adhered to a layer of woven fabric using a 100 micron TPF in combination with applying pressure and heat at 90˚ C for 10 seconds by heat press forming a 3-layer sandwich. This was done to cancel the stretchability effect due to the loop structure of the knit fabrics which leads to an increase in its resistance values when the loops' sizes increase due to stretching extension. In the next step, the sandwiched structure was cut by a laser cutting machine, resulting in the creation of a resistance value of 5 kΩ. However, as the conductive fabric is very lightweight the resulting structure was rather flimsy and difficult to handle, it was therefore, affixed to a paper tape on the conductive side. On the other side, the conductive fabric was bonded to a large section of woven base fabric by heat-pressing the structure with a 100 micron TPF. Finally, the tape is removed and the conductive side can be connected to circuits. The finished resistor prototype was 27 ×7.5 cm^2, which is a rather big surface area for a relatively small resistance (magnitude of few kΩs).

In the next set of samples, a target of creating small-sized resistors with sufficient resistance value was pursued. Therefore, for the main resistor part, CFY was picked due to its high resistance value. For the creation of the conductive transmission lines that are to be connected to circuits on both edges of the thin carbon yarn, Shieldex 235/36 dtex yarn was used.

In the design of sample R_{CFY1} design, a 22 cm CFY was attached to a base woven fabric by hand stitching. To achieve a higher resistance value in a compact form, the CFY was hand-stitched in a winding track pattern, allowing for a longer path within a smaller surface area, as shown in Figure 1 c), yielding a resistance value of 66 MΩ.

For sample R_{CFY2}, CFY yarn and Shieldex 235/36 dtex were once again used for resistance and transmission, respectively. The main CFY part was used in conjunction with Kevlar Ne16/2 which is a lightweight, heat-resistant yarn with a high tensile strength that enhances performance and protection, this aids in the support of the CFY yarn whose structure is very thin. Figure 1 d) illustrates how two knots were made on a distance of 7 mm on the CFY which was affixed to the Kevlar yarn using 12 cm of the Shieldex yarn, resulting in a 2.5 MΩ resistance value. To protect from external factors, the sample was covered with TPF and bonded by heat pressing it at 90° C heat for 20 seconds.

For the production of sample R_{CFY3}, a similar method to the one used to produce sample R_{CFY2} was used. CFY was once again employed for the main resistor part. As it has very high resistance (400 MΩ/m ± 40 MΩ/m), it requires cutting a very small distance of 2.5 mm to achieve a resistance of 1 MΩ. This small distance however makes the knotting of the transmission lines on the edges very challenging. Therefore an alternative design would be to connect two larger resistors in parallel. Thus, larger resistors were created by utilizing 5mm CFY in the same manner and connected

through their transmission line in parallel. TPU-coated yarn was used for transmission lines. After knotting the transmission line, the resistance was cut. To enable connection to circuits 3 cm of TPU is removed from the edge of the transmission lines. Finally, the prepared resistor components were placed between TPF to ensure isolation and structure stability. The two single and parallel designs are shown in Figure 1 e).

Finally, a resistor (R_{CFY4}) of value 90 KΩ was produced as described in the main text.

As for capacitor samples, Table 2 summarizes the materials and methods used in their production.

Table.2 Summary of the produced capacitors samples methods and materials.

**Sample Number**	**Production method**	**Materials**	**Electrical surface resistance Ω/□**	**Machines**	**Value**
**Sample $C_{T\text{-}tex}$**	Folding and gluing	-Conductive fabric: Shieldex Technic-tex P130 + B - Material.<br><br>-dielectric layer: FL120VS Polyethylene vacuum nylon 75 microns thickness.<br><br>-adhesion material: glue	<2 Ω/□	-for cutting conductive fabric: Universal laser systems VLS cutting machine.	7 nF
**Sample $C_{B\text{-}RS1}$**	Folding and applying heat and pressure	- Conductive fabric: Shieldex Bremen RS, woven fabric metallized with pure silver.<br><br>-dielectric layer:100 micron TPF	< 0.3 Ω/□	-for pressing fabric and vacuum nylon: Heat Press Model 1702	33 nF

**Sample $C_{B\text{-}RS2}$**	Folding and applying heat and pressure	- Conductive fabric: Shieldex Bremen RS, woven fabric metalized with pure silver.<br><br>-dielectric layer:100 micron TPF	< 0.3 Ω/□	-for pressing fabric and vacuum nylon: Heat Press Model 1702	1 nF
**Sample $C_{B\text{-}RS3}$**	Folding and applying heat and pressure	- Conductive fabric: Shieldex Bremen RS, woven fabric metalized with pure silver.<br><br>-dielectric layer: 8 micron Linear Low-Density PolyEthylene (LLDPE) stretch film.<br>-adhesive film for other layers: 100 micron TPF.	< 0.3 Ω/□	-for pressing fabric and vacuum nylon: Heat Press Model 1702	14 nF

Terada et al. [35] designed an electrode with an approximate capacitance value of 3 nF. Taking inspiration from their study with the folding method, the initial capacitor sample $C_{T\text{-}tex}$ design was crafted. Shieldex Technic-tex conductive knitted fabric was used to create the conductive plates of the capacitor while FL120VS nylon film acted as a dielectric layer. The conductive fabric was cut into two pieces, each with a width of 5 cm and a length of 60 cm, and twenty-one pieces of FL120VS with dimensions of 54 mm^2 were cut by the laser cutting machine.

During the capacitor production, conductive fabric sections were folded multiple times, and FL120VS nylon pieces were placed in each fold to prevent them from having contact with the other conductive plate fabric. In each folding process, the fabric and nylon pieces were glued utilizing strong adhesion glue, while ensuring there was no air trapped between the layers to avoid instability in capacitance value. The fabric was folded ten times on each side, resulting in a folded area in the finished sample measuring 5.5 × 5.5 × 1.5 cm^3 (width × height × depth) and a capacitance value of 7 nF.

For capacitor sample $C_{B\text{-}RS1}$, an alternative dimensionality needed to be achieved as sample $C_{T\text{-}tex}$ was rather bulky due to its thickness. For that purpose, the Shieldex Bremen RS conductive fabric was chosen for the conductive plates and 100-micron TPF was chosen for the dielectric layer. Two pieces of conductive fabric (60 × 10 cm^2) were cut as well as two pieces of TPFs (62 × 12 cm^2). On the cut TPF pieces, a 3 cm opening was created on the short edge of the TPF rectangle to create a conductive transmission area for attaching

crocodile clips during the electrical tests of the sample. The conductive and TPF pieces were adhered through a heat press in an alternating manner creating a sandwich. This sandwich structure was folded 6 times. Between each folding step, layers were heat pressed at 80° C for 7 seconds. Finally, the capacitance value was measured as approximately 33 nF, and the size was 12 × 8 × 0.25 cm^3 (width × depth × thickness). An important step in achieving a stable capacitance value was that special care needed to be taken to ensure that no air remained between the layers during the sealing process.

For sample $C_{B\text{-}RS2}$ (Figure 1 f)), the same conductive and dielectric materials utilized in sample $C_{B\text{-}RS1}$ were used. The conductive fabric layers were cut into dimensions of 6.5 × 5 cm^2. Placed in between those were the TPF layers measuring 9 × 6.5 cm^2. The arrangement consisted of TPF, the capacitor's 1st plate, TPF, the capacitor's 2nd plate and TPF respectively. It was heat pressed at 90° C for 20 seconds then folded one time and heat pressed again. TPU-covered yarn was stitched to the conductive plates after removing TPU from the contact point to facilitate connection to circuits.

Following those a final capacitor sample $C_{B\text{-}RS3}$ was made in the manner discussed in the main text.

Section 2: performance comparison between textile electrodes and Ag/AgCl gel electrodes.

To evaluate the efficacy of the textile electrodes' in ECG measurement, the performance of the produced electrodes was compared to the performance of the standard Ag/AgCl gel electrodes. An off-the-shelf AD8232 ECG conditioning circuit was used to record signals from both the textile and gel electrodes. Two types of activities were tested: static (sitting) and dynamic (twisting). A single female subject, who consented to the experiment, participated in the study. For each activity and electrode combination, data was recorded for 10 seconds. Figure2 presents the raw recorded signals, while Figure3 displays the signals after being digitally filtered to remove baseline wandering and powerline noise.

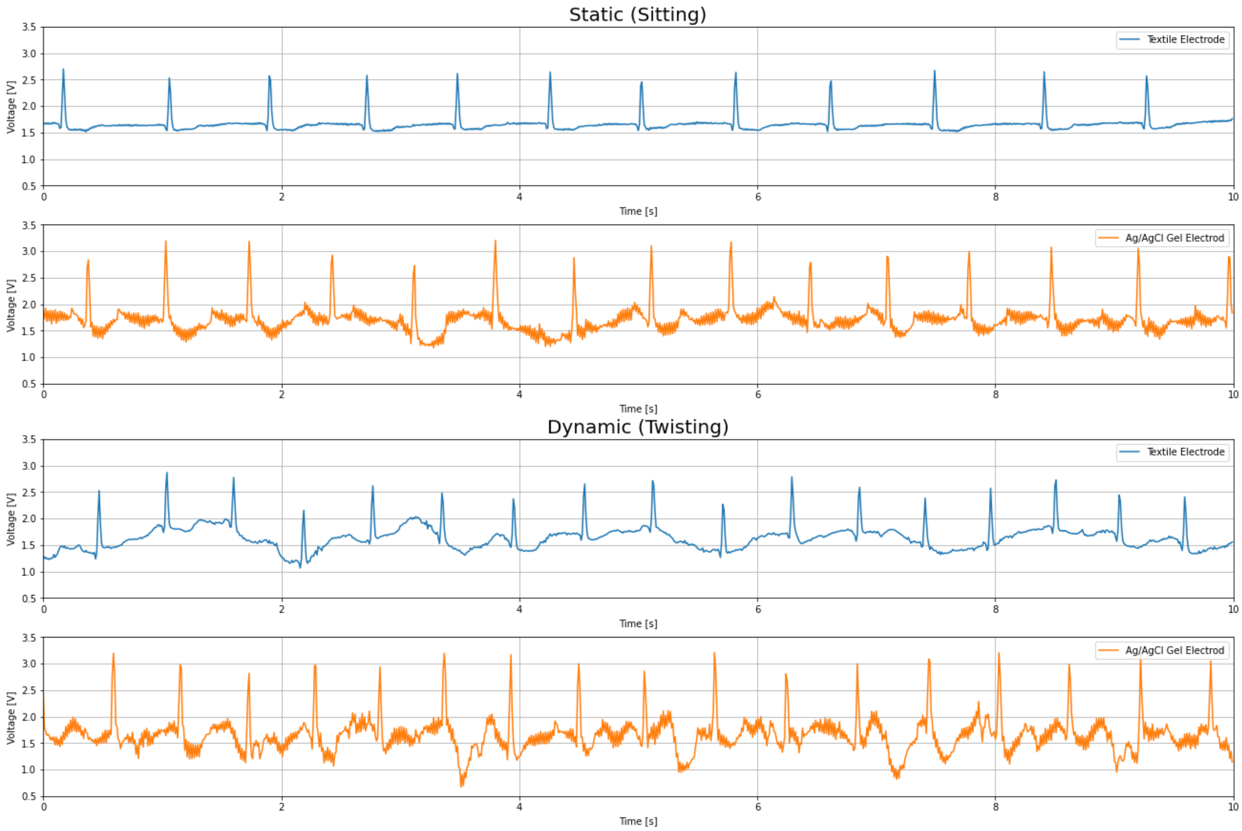

Figure 2: Ten seconds record of raw ECG signal using textile electrodes and Ag/AgCl gel electrodes for static and dynamic activities.

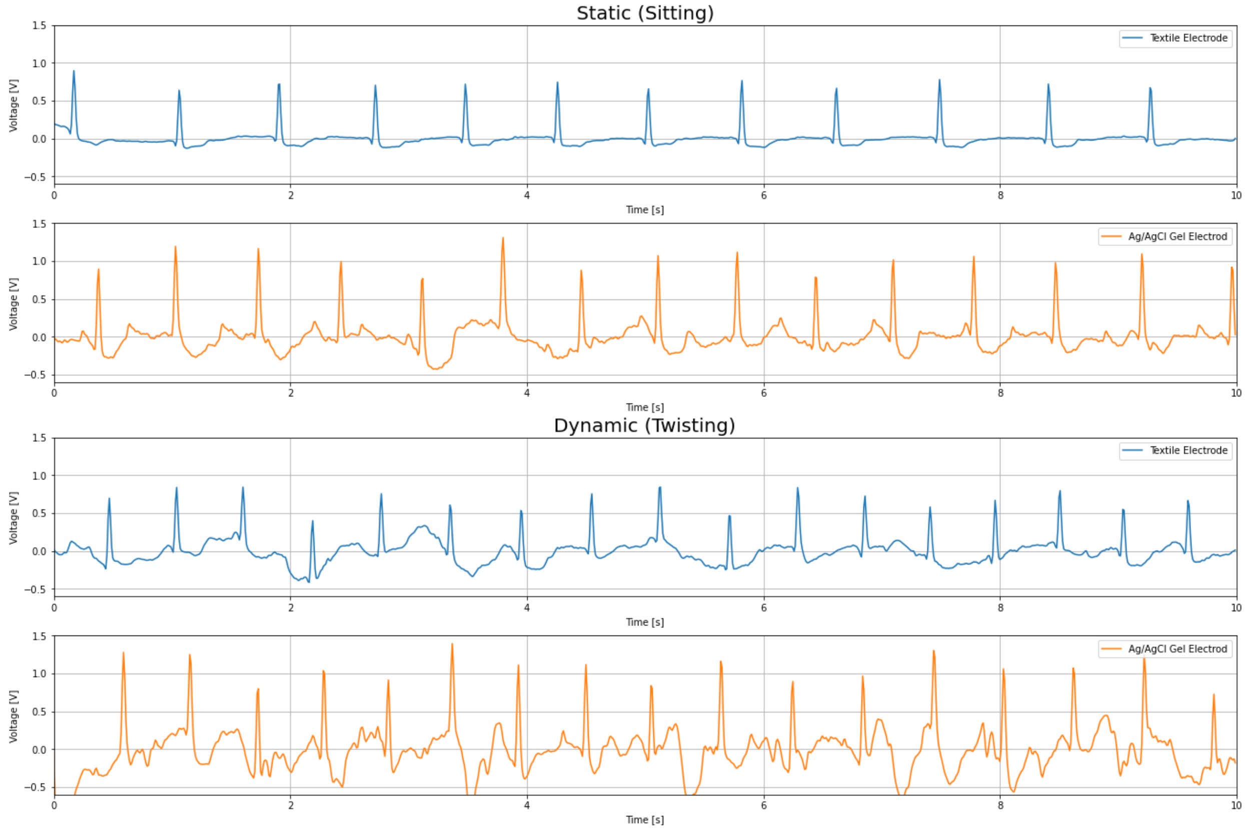

Figure 3: Ten seconds record of digitally filtered ECG signal using textile electrodes and Ag/AgCl gel electrodes for static and dynamic activities.

From Figures 2 and 3, it can be concluded that the signals recorded from gel electrodes exhibit higher voltage amplitude compared to those recorded from textile electrodes for both static and dynamic activities. However, textile electrodes demonstrate superior performance in terms of noise reduction and signal distortion. Specifically, signals captured with gel electrodes are more susceptible to power line noise. Even after applying digital filtering, fluctuations in the baseline between the peaks remain more pronounced in the gel electrode signals.

The Signal-to-Noise Ratio (SNR) of the signals was estimated using the method described in the main manuscript. The estimated average beat templates are shown in Figure 4, and the corresponding SNR results are provided in Table 3. The SNR estimation aligns with the visual findings presented in Figures 2 and 3.

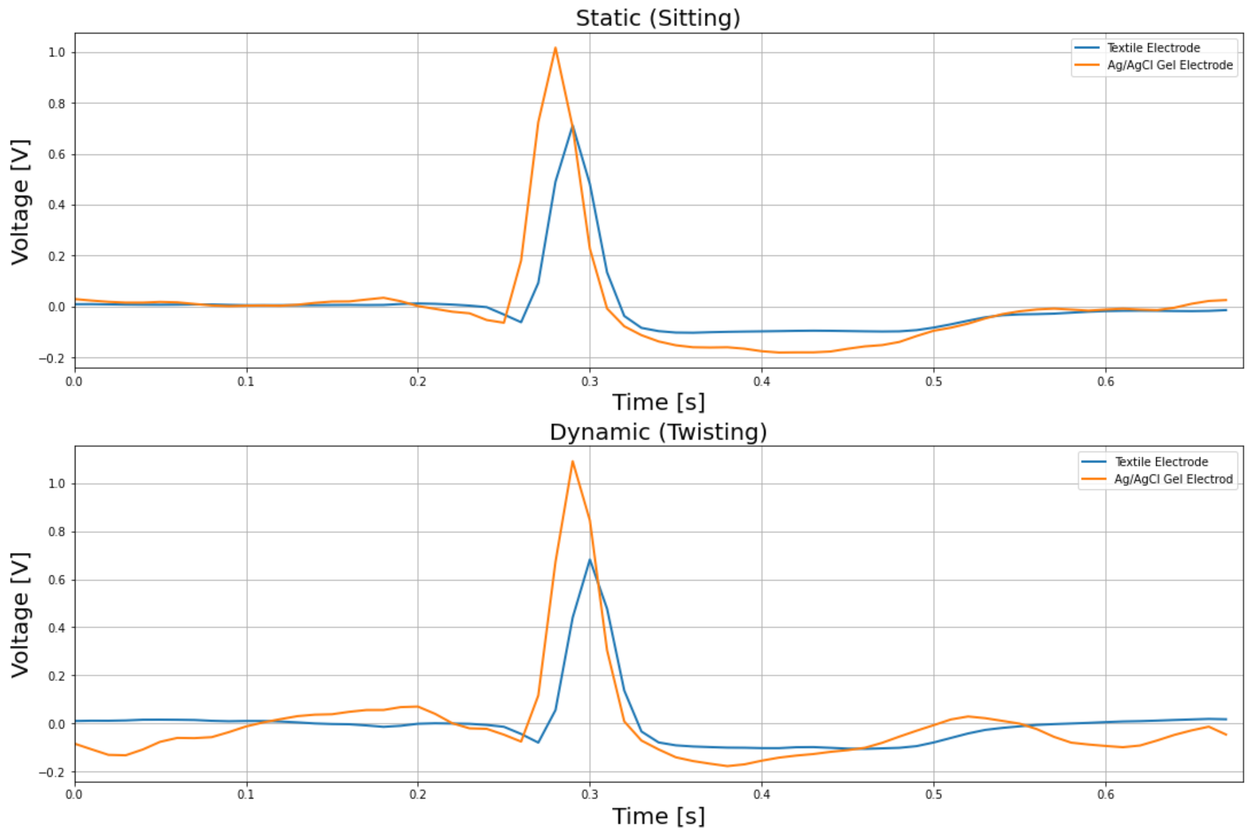

Figure 4. Estimated average beat template for the ten seconds recorded signals from the textile and the Ag/AgCl gel electrodes for both static and dynamic activities.

Table 3. Estimated SNR [dB] for the signals recorded using textile and Ag/AgCl electrodes

Activity Type	SNR using textile electrodes	SNR using Ag/AgCl gel electrodes
Static (sitting)	13.45	6.51
Dynamic (twisting)	4.85	1.13

Section 3: Subject-based bending test

Following the integration of the textile-based capacitors and resistors into the T-shirt, a bending test was conducted to simulate real-life conditions while the T-shirt was being worn. A single female subject, who consented to participate in the experiment, performed a series of activities designed to introduce a variety of bending and stretching motions in the chest area. Each activity was repeated 20 times to assess the impact of physical movement on the embedded components.

The first activity was a twisting motion. In this exercise, the subject stood with arms raised to chest level and elbows bent so that the fists hovered near the chest. She then twisted her torso to the left and returned to the right side, repeating the motion 20 times.

The second activity was chest extension. The subject stood straight with arms extended in front of her at shoulder level, elbows bent at 90 degrees, and fists raised in front of her face. She then opened her arms wide to each side, keeping the elbows and fists in a locked position, which caused the chest muscles to stretch. The subject then returned to the initial position and repeated the motion 20 times.

The third activity was bending down. The subject stood straight with arms extended in front of her at shoulder level, then bent at the waist while keeping the legs straight and the arms locked at the elbows. Once her arms were aligned with her legs and her palms touched near her ankles, she returned to the starting position. This motion was repeated 20 times.

As in the setup described in the main manuscript, a Keysight 34465A digital multimeter was used to continuously measure the values of the resistors and capacitors during the test. A program running on the PC collected the data throughout the experiment. The results of the bending test for each resistor and capacitor are shown in Figures 5-8, and the calculated tolerance values for each experiment are listed in Table 4.

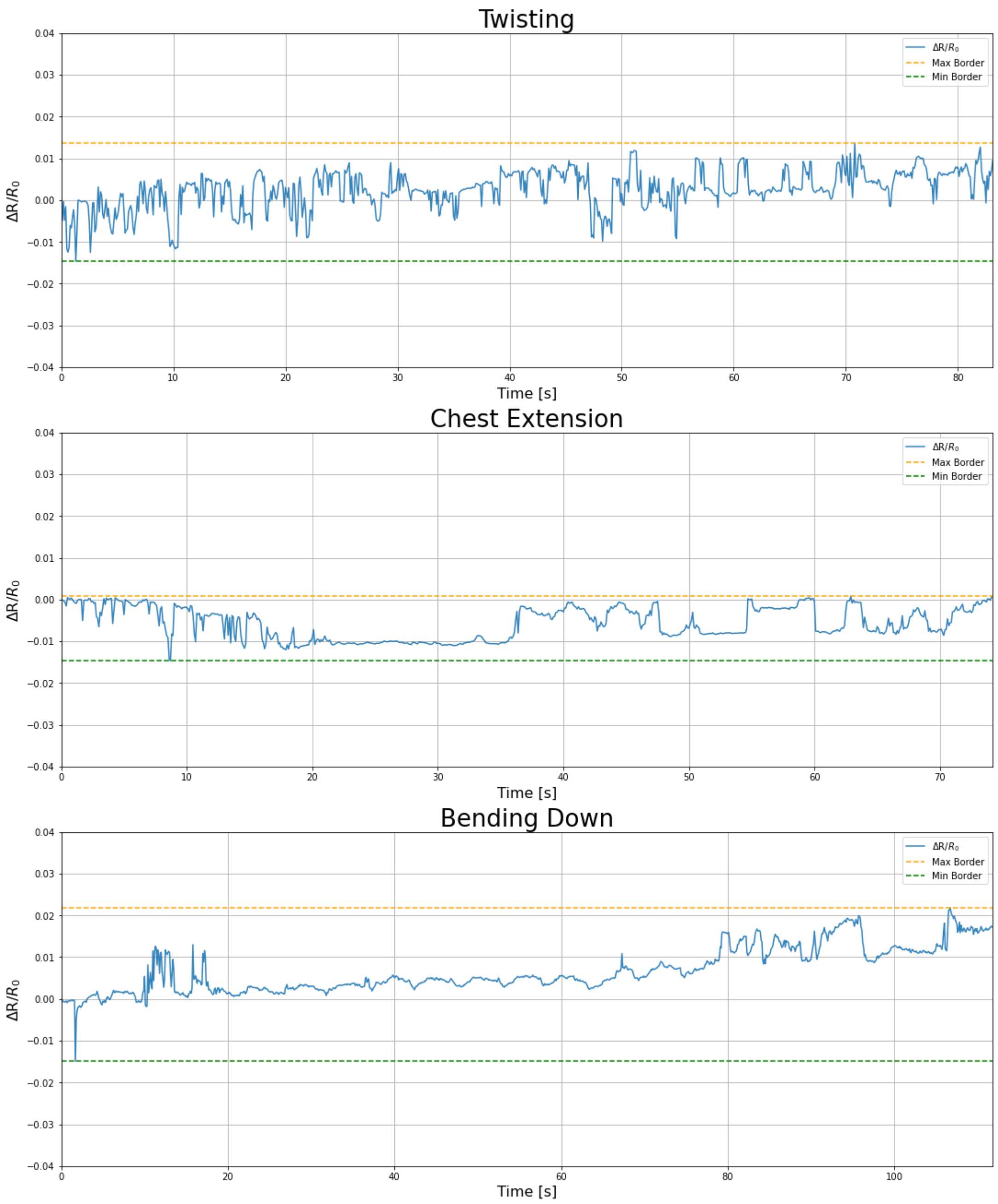

Figure 5. Resistance trend in multiple activities bending test with resistor sample 1.

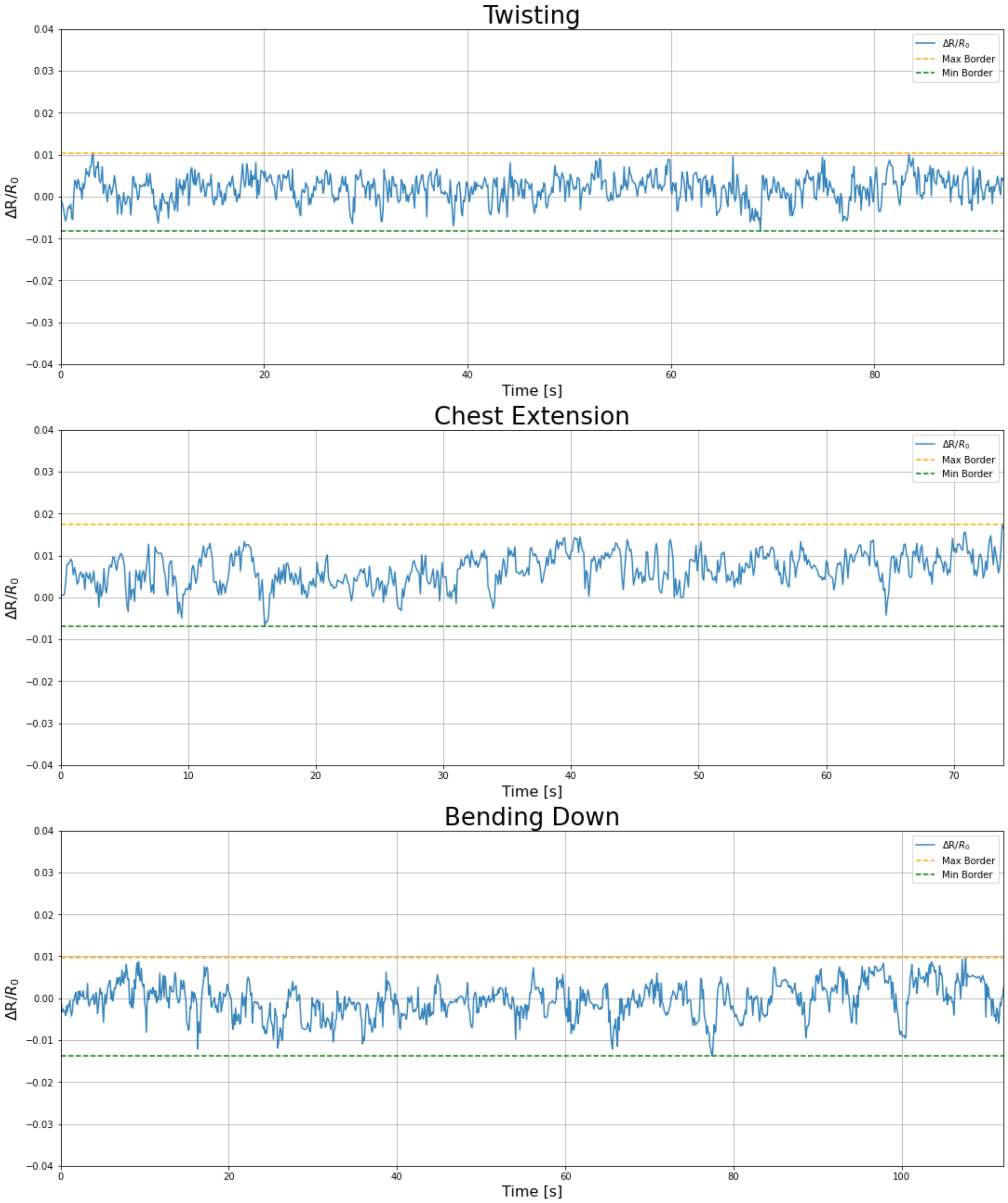

Figure 6. Resistance trend in multiple activities bending test with resistor sample 2.

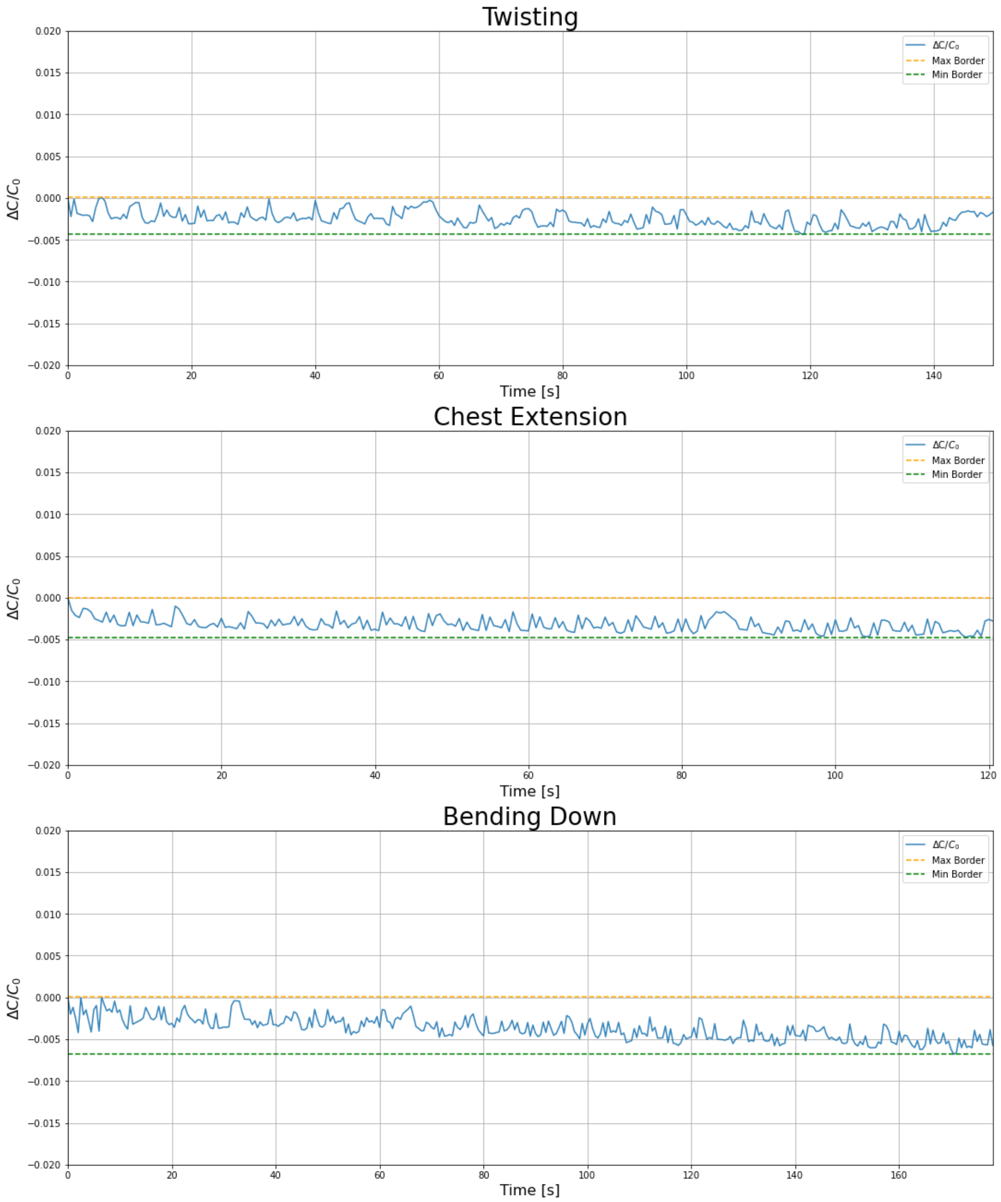

Figure 7. Capacitance trend in multiple activities bending test with capacitor sample 1.

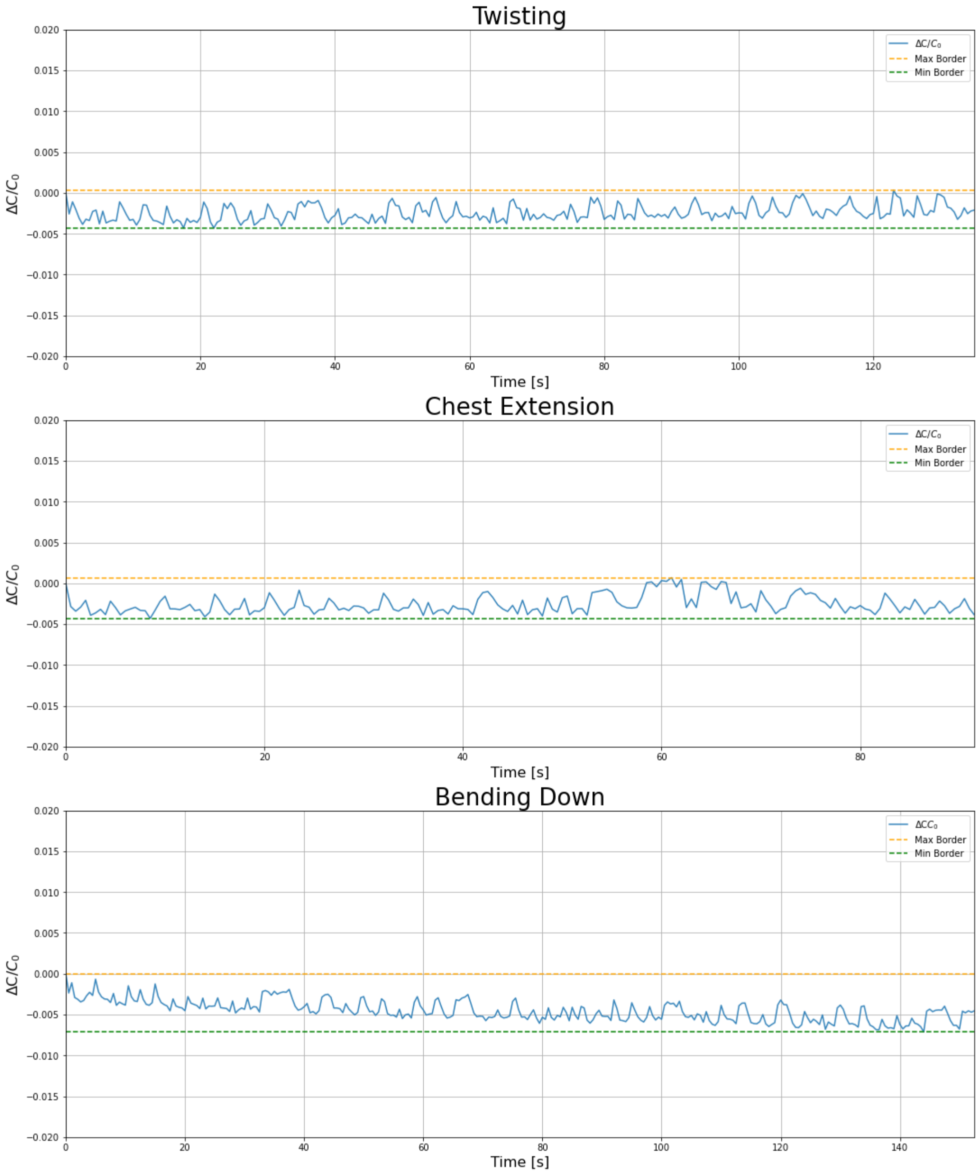

Figure 8. Capacitance trend in multiple activities bending test with capacitor sample 2.

Table 4: Tolerance results for bending tests with different activities.

Test	Twisting	Chest Extension	Bending Down	Average
R1	1.86%	2.44%	2.35%	2.22+/-0.25 %
R2	2.86%	1.57%	3.73%	2.72+/-0.88 %
C1	0.44%	0.47%	0.68%	0.53+/-0.10 %
C2	0.46%	0.50%	0.71%	0.56+/-0.11%

As shown in Table 4, it is confirmed that the average tolerance values for the resistor and capacitor samples during the tests with three activities were within the 5-10% range, which aligns with the tolerance value typically reported for commercial rigid resistors and capacitors.

Section 4: Comparison of related works

Table 5: Comparison of related works to the proposed work

Study	Targeted Application	Filtering type	Targeted Frequency band	Integration level	Technique and Material Type	R & C values	Dimensions	Measured filter performance metrics
[1]	Microwave Filter	Bandpass	High (7.58 GHz)	Full.	Textile based: embroidering conductive yarn on a textile substrate	N/A	few cm^2	Similarity of frequency response to simulation
[2]	Split Ring Resonators	Bandstop	High (2.7 GHz to 4.7 GHz)	Full.	Textile based: conductive yarn, cotton substrate	N/A	few cm^2	Similarity of frequency response to simulation
[3]	Filtering Antenna	Bandpass	High (2.5 GHz to 4.6 GHz)	Full.	inkjet printing a silver film on a textile substrate	N/A	37.5 mm × 23 mm × 0.49 mm	Similarity of frequency response to simulation
[4]	Patch Antenna	Bandpass	High (5.8GHz)	Full.	Textile based: weaving conductive fabric	N/A	40 mm x 40 mm	Similarity of frequency response to simulation
[5]	Capacitive ECG Electrode	RC High pass	Low	Partial: The textile electrode played the role of the capacitor and a standard ridged resistor was used with it.	Textile based: Conductive yarn	N/A	40×40 mm	Visual assessment of the signal quality

[6]	Capacitive ECG Electrode	RC High pass	Very Low (0.5 Hz–2 Hz)	Partial: The textile electrode played the role of the capacitor and a standard 3 GΩ ridged resistor was used with it.	Textile based: Conductive yarn	N/A	8 cm × 8 cm × 0.414 mm	Similarity of frequency response to simulation & Visual assessment of the signal quality
[7]	Capacitive ECG Electrode	Utilizing the body-to-electrode contact impedance as low pass RC filter	Low (100 Hz)	Partial: The textile electrode was created as a big capacitor to offset the contact impedance. No resistor was used.	Textile-based: conductive fabric, polyimide film	C=3.3nF	150 cm^2 fabric wrapped 7 times in 1 cm height	Similarity of frequency response to simulation & Visual assessment of the test signal & SNR estimation
[8]	RC Filter	RC Low pass	Medium (7 kHz - 1 MHz)	Full: both the capacitor and resistor were created.	inkjet printing Zn micro particle	R = 1.7 kΩ - 228 kΩ. C = 4.2 pF	Single body: 15mm × 60 mm	Assessment of frequency response
[9]	RC Filter	RC low pass and high pass	Low (100Hz - 800Hz)	Full: both the capacitor and resistor were created.	Inkjet printing conductive polymer	N/A	N/A	Visual assessment of the test signal
[10]12/1/2024 11:06:00 AM	Resistors and RC Filter	RC low pass	Low to medium (0.4 Hz to 27 kHz)	Full: both the capacitor and resistor were created.	Inkjet printing reactive silver ink	R= 12 KΩ - 800 KΩ C=0.43nF	N/A	Frequency response simulation & Visual assessment of the test signal
[11]	Radio frequency filter	CL low pass	High (2 GHz)	Full: both the capacitor and inductor were created.	Inkjet printing silver ink	C=2pF L= 8nH	C = 0.9 mm^2 L radius =4 mm	Similarity of frequency response to simulation

[12]	Resistors, capacitors, and inductors production	RC low pass and RL high pass	Low (160Hz) And medium (160KHz)	Full: the capacitor, resistor, and inductor were created.	Inkjet printing	R= 100 Ω C= 10μF L=10 μH	2 × 1.25 mm	Similarity of frequency response to simulation & Visual assessment of the test signal
[13]	RC filter	RC lowpass	Medium (100 KHZ)	Full both the capacitor and resistor, were created.	3D printing	R=80K Ω-160K Ω C=4.7pF-9.3pF	Few cm^2 per component	Similarity of frequency response to simulation
[14]	Microwave Filter	Lowpass filter	High (2.4GHz)	Full.	3D printing	N/A	20 × 39.5 mm	Similarity of frequency response to simulation
[15]	Split Ring Resonators	Band pass	High (1.6 and 2.45 GHz)	Full.	3D printing	N/A	0.035 × 20 mm	Similarity of frequency response to simulation
[16]	AC line filtering	Post-rectification ripple reduction	Low (10 -120 Hz)	Electro mechanical Capacitor was produced.	Wet spinning reduced graphene oxide fibers	N/A	N/A	Frequency responses assessment and visual assessment of the test signal
[17]	LC filter	Lowpass	Low (500 - 1000 Hz)	Full: the capacitor, inductor, and resistor were created.	Direct write 3D printing	R= 1k Ω C= 1.8pF L= 2.2 μH	R= 2.00× 1.25× 0.60mm C= 2.00 × 1.25 × 0.70mm L= 2.00× 1.25 × 1.80mm	Similarity of frequency response to commercial filter
[18]	LC filter	Lowpass T-topology	High (250 MHz)	Full: the capacitor and	3D printing	C=25.5pF L1=L2=3 1.8 nH	Totally encased in	Similarity of frequency response to simulation

				inductors were created.			19 × 19 × 32 mm3	
[19]	Wearable capacitor	N/A	N/A	The capacitor was created.	Inkjet printing silver ink on textile substrate	C=163 pF	7.07 mm^2	Comparison of frequency response that of commercial model when a resistance is connected in series and parallel to it.
[20]	Wearable Radio Frequency (RF) filter	Bandpass RF filter	High (2.4 GHz)	Full.	Screen printing on textile substrate	N/A	Few cm^2	Comparison of frequency response to simulation
The proposed work	RC Filter	RC Low pass	Low (100 Hz)	Full: both the capacitor and resistor are created utilizing textile-based materials.	Textile based: conductive yarn, conductive fabric, Thermoplastic film	R= 90 k Ω<br>C=14 nF	R = 2 × 3 cm^2<br>C= 8 ×8 cm^2	Similarity of frequency response to simulation &<br>Visual assessment of the test signal<br>& SNR estimation

References:

[1] B. Moradi, R. Fernández-García, I. Gil, *Electronics* **2018**, *8*, 11.

[2] B. Moradi, R. Fernández-García, I. G. Gali, *Applied Sciences* **2021**, *11*, 10930.

[3] H.-L. Kao, C.-H. Chuang, C.-L. Cho, in *2019 IEEE 69th Electronic Components and Technology Conference (ECTC)*, IEEE, Las Vegas, NV, USA, **2019**, pp. 258–263.

[4] M. I. Ahmed, M. F. Ahmed, A.-E. H. Shaalan, *PIER C* **2018**, *83*, 255.

[5] H. Li, X. Chen, L. Cao, C. Zhang, C. Tang, E. Li, X. Feng, H. Liang, *Transactions of the Institute of Measurement and Control* **2017**, *39*, 141.
[6] B. Babusiak, S. Borik, L. Balogova, *Measurement* **2018**, *114*, 69.
[7] T. Terada, M. Toyoura, T. Sato, X. Mao, *Sensors* **2021**, *21*, 4305.
[8] A. Radwan, Y. Sui, C. A. Zorman, *Flex. Print. Electron.* **2024**, *9*, 025001.
[9] B. Chen, T. Cui, Y. Liu, K. Varahramyan, *Solid-State Electronics* **2003**, *47*, 841.
[10] M. Cao, K. Jochem, W. J. Hyun, L. F. Francis, C. D. Frisbie, *Flex. Print. Electron.* **2018**, *3*, 045003.
[11] G. McKerricher, M. Vaseem, A. Shamim, *Microsyst Nanoeng* **2017**, *3*, 16075.
[12] P. Lall, K. Goyal, S. Miller, in *2022 21st IEEE Intersociety Conference on Thermal and Thermomechanical Phenomena in Electronic Systems (iTherm)*, IEEE, San Diego, CA, USA, **2022**, pp. 1–10.
[13] P. Esposito, G. Barile, V. Stornelli, G. Ferri, in *2024 9th International Conference on Smart and Sustainable Technologies (SpliTech)*, IEEE, Bol and Split, Croatia, **2024**, pp. 1–5.
[14] U. Robles, E. Bustamante, P. Darshni, R. C. Rumpf, *PIER M* **2019**, *84*, 147.
[15] A. Vallecchi, D. Cadman, W. G. Whittow, J. Vardaxoglou, E. Shamonina, C. J. Stevens, *IEEE Trans. Microwave Theory Techn.* **2019**, *67*, 4341.
[16] K. Gao, S. Wang, W. Liu, Y. Yue, J. Rao, J. Su, L. Li, Z. Zhang, N. Liu, L. Xiong, et al., *ChemElectroChem* **2019**, *6*, 1450.
[17] P. Lall, J. Narangaparambil, K. Schulze, S. Miller, in *2021 20th IEEE Intersociety Conference on Thermal and Thermomechanical Phenomena in Electronic Systems (iTherm)*, IEEE, San Diego, CA, USA, **2021**, pp. 787–796.
[18] J. M. Lopez-Villegas, N. Vidal, A. Salas, *Additive Manufacturing* **2021**, *48*, 102410.
[19] Y. Li, R. Torah, S. Beeby, J. Tudor, in *2012 IEEE Sensors*, IEEE, Taipei, Taiwan, **2012**, pp. 1–4.
[20] S. An, A. Meredov, A. Shamim, in *2018 48th European Microwave Conference (EuMC)*, IEEE, Madrid, **2018**, pp. 831–834.
